# Structure–Activity Relationship Studies of Pyrrolone Antimalarial Agents

**DOI:** 10.1002/cmdc.201300177

**Published:** 2013-08-05

**Authors:** Dinakaran Murugesan, Marcel Kaiser, Karen L White, Suzanne Norval, Jennifer Riley, Paul G Wyatt, Susan A Charman, Kevin D Read, Clive Yeates, Ian H Gilbert

**Affiliations:** [a] Division of Biological Chemistry & Drug Discovery, College of Life Sciences, University of DundeeSir James Black Centre, Dow St, Dundee, DD1 5EH (UK); [b] Swiss Tropical & Public Health Institute, University BaselPostfach, Socinstrasse 57, 4002 Basel (Switzerland), Petersplatz 1, 4003 Basel (Switzerland); [c] Centre for Drug Candidate Optimisation, Monash Institute of Pharmaceutical Sciences, Monash University381 Royal Parade, Parkville, Victoria 3052 (Australia); [d] InPharma ConsultancyHertfordshire (UK)

**Keywords:** antiprotozoal agents, malaria, *Plasmodium falciparum*, pyrrolones, structure–activity relationships

## Abstract

Previously reported pyrrolones, such as TDR32570, exhibited potential as antimalarial agents; however, while these compounds have potent antimalarial activity, they suffer from poor aqueous solubility and metabolic instability. Here, further structure–activity relationship studies are described that aimed to solve the developability issues associated with this series of compounds. In particular, further modifications to the lead pyrrolone, involving replacement of a phenyl ring with a piperidine and removal of a potentially metabolically labile ester by a scaffold hop, gave rise to derivatives with improved in vitro antimalarial activities against *Plasmodium falciparum* K1, a chloroquine-and pyrimethamine-resistant parasite strain, with some derivatives exhibiting good selectivity for parasite over mammalian (L6) cells. Three representative compounds were selected for evaluation in a rodent model of malaria infection, and the best compound showed improved ability to decrease parasitaemia and a slight increase in survival.

## Introduction

Malaria is a serious disease endemic in tropical and subtropical regions of the world. It is a major threat to public health in more than 100 countries and is responsible for >200 million clinical cases each year and probably about 1 million deaths, the majority of whom are young children and pregnant women.[1–4] In addition, malaria is responsible for a huge economic impact in endemic countries.[2] With increasing globalisation and global warming, there is a risk of malaria spreading to new areas.[3] No vaccine is currently available for malaria, and the resistance of the protozoa to clinically used chemotherapeutic agents is increasingly common. Therefore, an urgent need exists to develop new classes of antimalarial drugs that operate by novel mechanisms of action.

**Figure 1:**
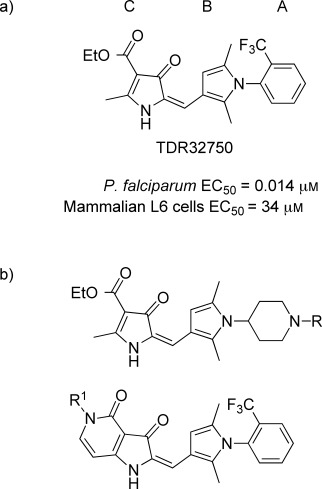
a) Prototypical compound TDR32750, structure and in vitro activities.[5] b) Generic structure of compounds prepared in this study.

We have previously reported on the discovery and structure–activity relationship (SAR) of a series of pyrrolones, which have potent antimalarial activity;[5] these were initially discovered through a phenotypic screening process conducted by the World Health Organisation (WHO) Programme for Research and Training in Tropical Diseases. The prototypical compound TDR32750 (Figure 1 a) showed potent antimalarial activity against a panel of strains of *Plasmodium falciparum* and significant depression of parasitaemia (>99 %) in the *Plasmodium berghei* mouse model of malaria when dosed intraperitoneally. Unfortunately, the compound has low oral bioavailability; good oral bioavailability is a requirement for most target product profiles for malaria. While the precise reason for the low bioavailability is unknown, it is most likely due to a combination of metabolic instability (as evidenced by studies in hepatic microsomes) and low aqueous solubility.[5] The ester linkage is also susceptible to hydrolysis in blood, which contributes to the overall systemic clearance, as previously reported. Our previous SAR studies around TDR32750[5] encompassed changes to the A-, B-and C-rings (Figure 1 a), which led to the conclusion that the A-ring was reasonably tolerant of changes, unlike the B-ring. Modifications to the C-ring were not investigated apart from replacement of the ester with a variety of amides, which led to compounds with decreased activity.

In order to improve the physicochemical properties of the molecules and the prospects for further development of the compound series, our aim was to decrease the *c*log D value to less than three. Here, we report on changes to the A-ring and C-ring, which have led to compounds with enhanced antimalarial activity and improved physicochemical properties. In particular, our work focused around some piperidine analogues of the A-ring in our lead molecule (Figure 1 b). The piperidine moiety has an amino group, which when unconjugated is basic and should improve the solubility properties.

The lead compound, TDR32750, contains an ester in the C-ring; we were concerned that this might be a metabolic liability due to hydrolysis by esterases, since previous work has confirmed that there is gradual degradation of the ester in vivo.[5] However, this is probably not the only mode of metabolism, as we have also shown that the compound undergoes cytochrome P450 (CYP450)-mediated degradation in hepatic microsomes.[5] Nevertheless, we thought it prudent to try and replace the ester to improve the clearance properties. Previously, we reported efforts at replacing the ester with an amide, but this led to loss of activity; possibly due to the amide causing a conformational change compared to the ester.[5] In this paper, we report the effect of cyclising the ester to form a fused pyridone, which might lock the compound into a more favourable conformation (Figure 1 b).

## Results and Discussion

### Chemistry

**Scheme 1:**
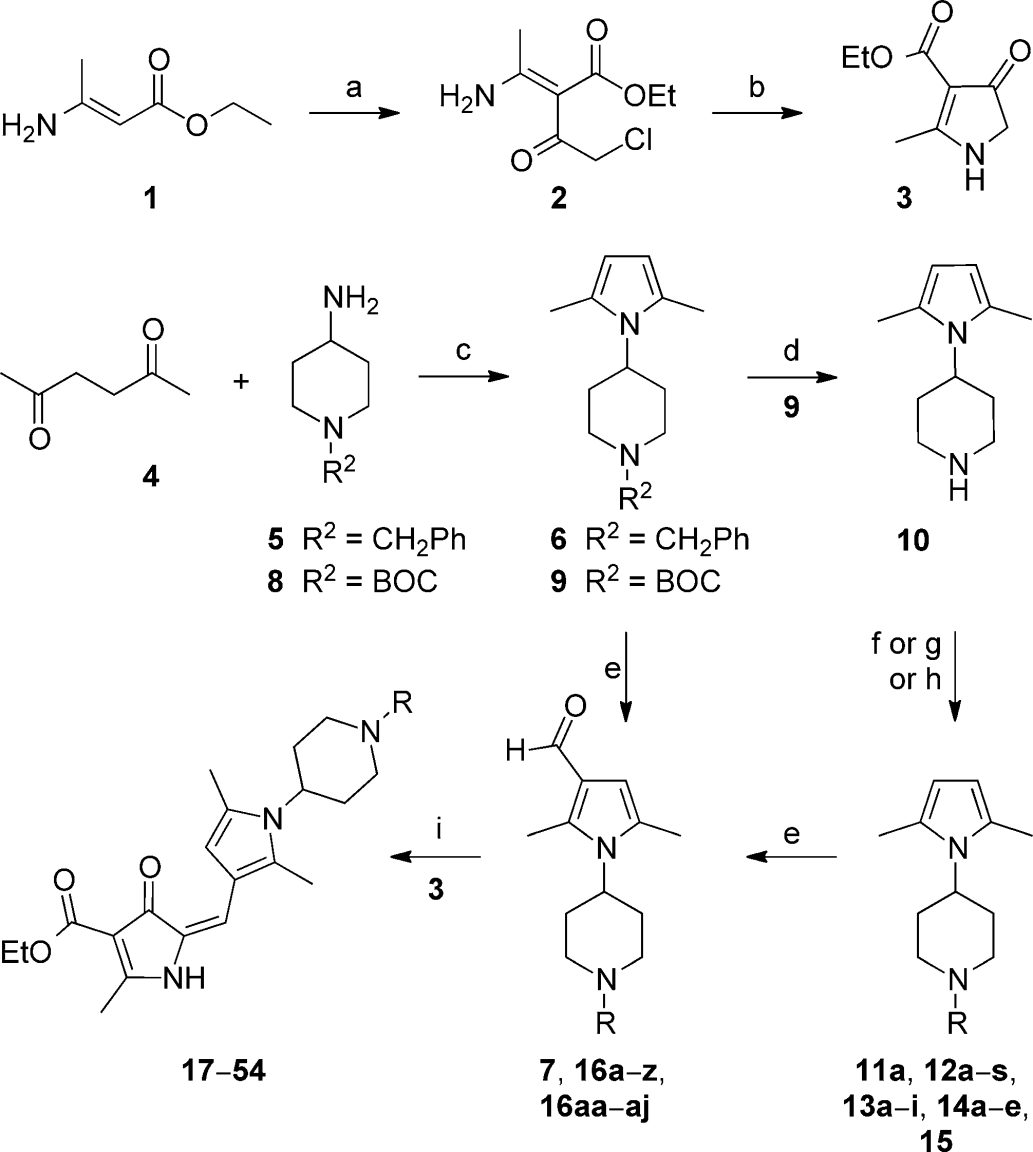
General strategy for the synthesis of piperidine derivatives. *Regents and conditions*: a) chloroacetyl chloride, pyridine, 0 °C, 30 min, 75 % yield; b) KOH, EtOH, 3 h, 0 °C, 95 % yield; c) *p*-TsOH bound on silica gel, microwave (0–400 W at 2.45 GHz), 180 °C, 15–20 min, 80–90 % yield; or *p*-TsOH, toluene, Dean–Stark apparatus, 90 °C, 3 h, 80–90 %; d) 4 m HCl/dioxane, 0 °C, 3 h, 66 % yield; e) POCl_3_, DMF, 100 °C, 3 h, 80–95 % yield; f) ROSO_2_CH_3_, Et_3_N, NaHCO_3_, CH_3_CN, 85 °C, 12 h, 50–70 %; g) RBr, DIPEA, DMF, 85 °C, 3 h, 40–70 % yield; h) alkyl/aryl/heterocyclic aldehyde, sodium triacetoxyborohydride, MeCN, RT, 8 h, 60–80 % yield; i) KHSO_4_, EtOH, 3 h, reflux, 80–95 % yield. R groups are given in Table 1.

The starting point for the synthesis of the A-ring variants incorporating a piperidine substituent was pyrrolone **3**, which was prepared as reported previously (Scheme 1).[5] Pyrrolone **3** (which was not stored but used immediately) was then condensed with the required 3-formylpyrrole 4-aminopiperidine (**7**, **16 a**–**z**, **16 aa**–**aj**) in the presence of potassium hydrogen sulfate,[6], [7] to give predominantly the (*E*)-isomer of the requisite pyrrolone derivative (**17**–**54**).^1^

3-Formylpyrrole intermediates **16 a**–**z** and **16 aa**–**aj** were prepared in a four-step sequence: 1) condensation of *tert*-butyloxycarbonyl (BOC)-protected 4-aminopiperidine (**8**) with 2,5-hexandione **4** (Paal–Knorr pyrrole synthesis); 2) deprotection of the BOC group; 3) coupling either by alkylation or reductive amination to give a range of piperidine derivatives (**11 a**–**15**); 4) carbonylation using a Vilsmeier–Haack reaction (85–95 % yield).[8], [9] The complete range of compounds prepared is shown in Table 1.

**Scheme 2:**
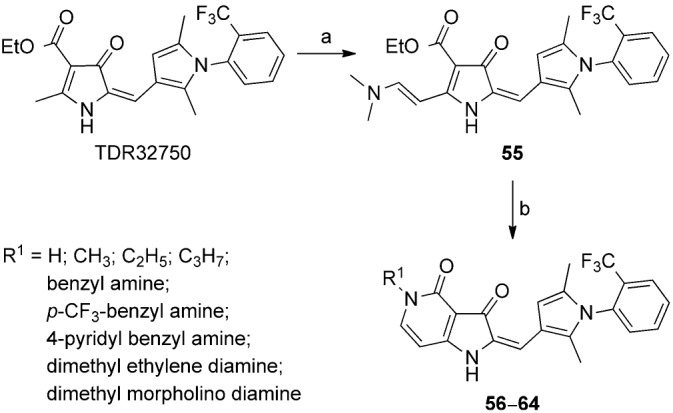
a) DMF–diethyl acetal, 85 °C, 15 min, 100 % yield; b) R^1^NH_2_, *iso-*propyl alcohol, microwave (0–400 W at 2.45 GHz), 110 °C, 15–30 min, 30–70 % yield.

The pyrrolo [3,2-*c*] pyridine-3, 4-dione series, that is the C-ring analogues of TDR32750 incorporating a fused pyridone ring, were prepared according to Scheme 2. Reaction of the pyrrolone ring of TDR32750 with the diethyl acetal of *N*,*N*-dimethylformamide (DMF) gave key intermediate **55.**[10] Reaction with an amine caused displacement of the dimethylamine and cyclisation to the required pyrrolo[3,2-*c*] derivatives **(56**–**64**).

### In vitro activity

Compounds were evaluated for activity against *P. falciparum* K1, a chloroquine-and pyrimethamine-resistant parasite strain, and counter-screened against mammalian L6 cells (Table 1). Many of the compounds showed potent antimalarial activity, with eight compounds showing EC_50_ values of less than 10 nm and two compounds (**29** and **44**) showing sub-nanomolar potencies. Most of the compounds showed good selectivity for parasite over mammalian cells.

**Table 1:** Activity data for A-ring variants against *Plasmodium falciparum*.

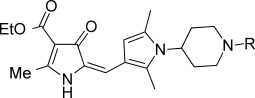								
Compd[a]	R	EC_50_[b] [μm]		*c*Log D[c]	Solubility[d] [μm]			CL_int_[e]
		*P. falc.*	L6 cells	(pH 7.4)	pH 2.0	pH 6.5	water	[μL min^−1^ mg^−1^]
TDR32750		0.014	34	5.0	15–30	7–15	>125	50^#^
**17**	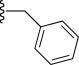	0.035	20	2.1	56–110	14–28	>125	60^#^
**18**	-H	12	>250	0.7	n.d.	n.d.	n.d.	n.d.
**19**	-CH_3_	1.4	7.8	0.9	n.d.	n.d.	n.d.	n.d.
**20**	-C_2_H_5_	2.4	7.6	1.2	>250	>250	n.d.	11^#^
**21**	-(CH_2_)_2_C(CH_3_)_2_	0.19	22	2.0	n.d.	n.d.	n.d.	n.d.
**22**	-(CH_2_)_2_CH_3_	1.1	6.6	1.4	n.d.	n.d.	n.d.	n.d.
**23**	-CH_2_C(CH_3_)_3_	0.058	88	2.0	n.d.	n.d.	n.d.	n.d.
**24**	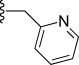	0.38	5.9	1.6	n.d.	n.d.	n.d.	n.d.
**25**	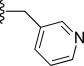	0.46	27	1.6	n.d.	n.d.	n.d.	n.d.
**26**	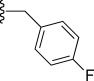	0.024	51	2.2	n.d.	n.d.	>125	117^#^
**27**	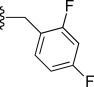	0.023	23	2.2	n.d.	n.d.	>125	149^#^
**28**	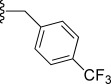	0.002	5	2.7	48-97	6–12	>125	217^§^
**29**	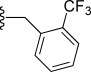	0.0005	1.7	2.6	n.d.	n.d.	>125	952^§^
**30**	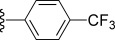	0.024	10	2.7	n.d.	n.d.	>125	n.d.
**31**	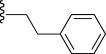	0.32	107	2.2	n.d.	n.d.	n.d.	76^#^
**32**	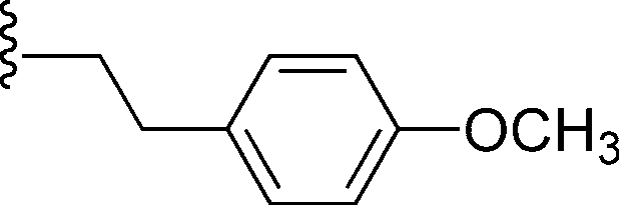	0.083	72	2.2	100–200	25–51	n.d.	120^#^
**33**	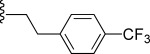	0.13	19	2.6	n.d.	n.d.	n.d.	215^#^
**34**	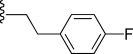	0.096	22	2.3	52–100	13–26	n.d.	114^#^
**35**	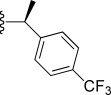	0.007	4.26	2.9	n.d.	n.d.	n.d.	88^§^
**36**	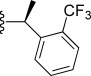	0.001	3	2.9	n.d.	n.d.	>125	190^§^
**37**	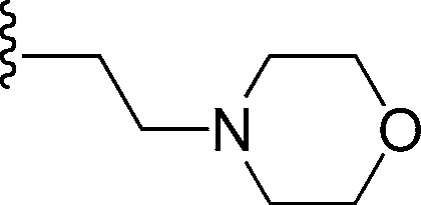	5.3	89	1.3	n.d.	n.d.	n.d.	n.d.
**38**	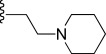	2.4	97	1.5	n.d.	n.d.	n.d.	n.d.
**39**	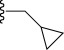	3.5	14	1.7	n.d.	n.d.	n.d.	n.d.
**40**	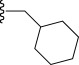	0.016	13	2.1	n.d.	n.d.	>125	78^§^
**41**	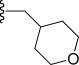	0.11	43	1.6	n.d.	n.d.	n.d.	n.d.
**42**	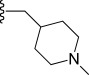	1.4	81	1.4	n.d.	n.d.	n.d.	n.d.
**43**	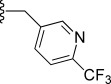	0.004	49	2.2	n.d.	n.d.	>125	177^§^
**44**	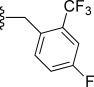	0.0004	1.2	2.6	n.d.	n.d.	>125	248^§^
**45**	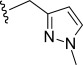	0.15	36	1.2	n.d.	n.d.	n.d.	n.d.
**46**	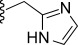	1.2	28	1.1	n.d.	n.d.	n.d.	n.d.
**47**	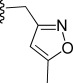	0.10	42	1.5	n.d.	n.d.	n.d.	n.d.
**48**	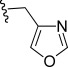	0.018	0.16	1.3	n.d.	n.d.	>125	<10^§^
**49**	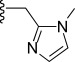	1.0	16	1.0	n.d.	n.d.	n.d.	n.d.
**50**	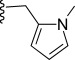	0.012	3	1.4	n.d.	n.d.	n.d.	55^§^
**51**	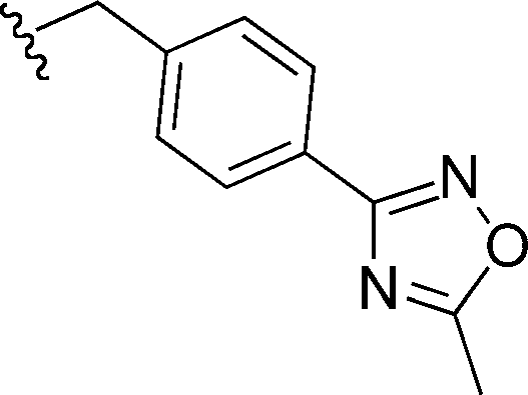	0.001	11	2.4	n.d.	n.d.	>125	n.d.
**52**	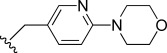	1.5	50	2.2	n.d.	n.d.	n.d.	n.d.
**53**	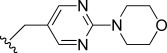	0.075	28	1.8	n.d.	n.d.	n.d.	n.d.
**54**	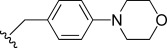	2.6	53	2.5	n.d.	n.d.	n.d.	n.d.

[a] Overall yields: 40–85 %; reference compounds: chloroquine, EC_50_=0.095–0.172 μm (*P. falciparum* K1); podophyllotoxin, EC_50_=0.009–0.022 μm (L6 cells).

[b] The EC_50_ values are the mean of two independent assays, which varied less than ±50 %; n.d=not determined.

[c] Calculated using StarDrop (http://www.optibrium.com).

[d] Measured using nephelometry.

[e] Intrinsic clearance determined in vitro using mouse liver microsomes at Monash University (#) or at the University of Dundee (UK) (§).

#### A-Ring modifications

The following observations were made regarding the in vitro antimalarial activity for compounds with variations around the A-ring:

- In general, simple alkyl substituents (**18**–**23**) led to compounds that exhibit a large decrease in activity compared with TDR32750. This also applied to a methylene cyclopropyl derivative (**39**). Interestingly, methylene cyclohexyl derivative **40** showed only a slight drop in activity compared with TDR32750, although adding heteroatoms to the ring to improve the physicochemical properties of the compounds was counter-productive for activity (**41** and **42**). Similarly, cyclohexyl analogues linked via an ethylene linker (**37** and **38**) showed decreased activity.
- Where the substituent was an aromatic ring directly attached to the piperidine ring (**30**), the compound retained reasonably potent activity (EC_50_=0.024 μm); however, this compound would have decreased basicity at the piperidine ring.
- Compounds with a phenyl ring separated from the piperidine by a methylene linker in general showed good potency (**17**, **26**–**29**, **35**, **36**, **43**, **44**, **51**).
- Replacement of the phenyl ring by a heteroaromatic ring (to improve physicochemical properties) was generally detrimental to activity (**24**, **25**, **45**–**47**, **49**, **52**, **53**). The marked exception to this was compound **43**, a pyridine, albeit with an electron-withdrawing trifluoromethyl group, which is likely to significantly decrease the basicity of the pyridine nitrogen. The oxazole (**48**) and *N*-methylpyrrole (**50**) derivatives also retained activity.
- Increasing the spacer length between the piperidine and the aromatic ring also led to a drop in activity (**31**–**34**).
- The phenyl ring was tolerant to different substitutions, although the morpholine analogue (**54**) had greatly decreased activity.

#### C-Ring modifications

Modifications to the C-ring showed interesting activities in vitro against *P. falciparum* K1 (Table 2). The most active compounds displayed sub-micromolar activities; compounds **56**, **57**, **58** and **63** were found to be the most active. The following observations were made regarding the SAR of the C-ring-modified analogues:

**Table 2:** Activity data for C-ring variants against *Plasmodium falciparum*.

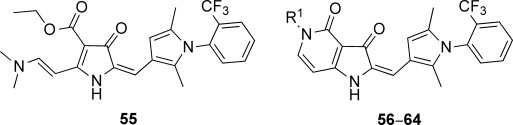						
Compd[a]	R^1^	EC_50_[b] [μm]		*c*Log D[c]	Solubility [μm]	CL_int_[d]
		*P. falc.*	L6 cells	(pH 7.4)	water	[μL min mg^−1^ protein]
**55**	–	0.061	56	2.8	>125	n.d.
**56**	H	0.047	54	4.0	>125	69
**57**	CH_3_-	0.014	29	4.2	>125	286
**58**	CH_3_CH_2_	0.021	36	4.5	>125	381
**59**	CH_3_CH_2_CH_2_	0.15	34	4.7	n.d.	n.d.
**60**	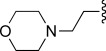	0.23	65	2.8	n.d.	n.d.
**61**	(CH_3_)_2_NCH_2_CH_2_-	0.25	35	2.4	n.d.	n.d.
**62**	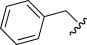	0.23	43	5.5	n.d.	n.d.
**63**	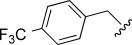	0.046	54	5.9	89	n.d.
**64**	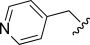	0.077	38	4.5	>125	n.d.

[a] Overall yields: 50–90 %; reference compounds: chloroquine, EC_50_=0.019–0.066 μm (*P. falciparum* K1); podophyllotoxin, EC_50_=0.012 μm (L6 cells).

[b] The EC_50_ values are the mean of two independent assays, which varied less than ±50 %; n.d=not determined.

[c] Calculated using StarDrop (http://www.optibrium.com).

[d] Intrinsic clearance determined in vitro using mouse liver microsomes at the University of Dundee (UK).

- Compound **56** with a free NH group on the pyrrolo[3,2-*c*]pyridine ring showed an EC_50_ value of 0.047 μm. Small alkyl substituents on the “pyridone” nitrogen were tolerated (**57**, R=Me: EC_50_=14 nm; **58**, R=Et: EC_50_=21 nm), whilst a propyl substituent (**59**) led to a tenfold drop in activity.
- Basic substituents did not appear to be tolerated, with compounds (**60** and **61**) exhibiting approximately a fivefold decrease in EC_50_ value relative to the unsubstituted analogue **56**.
- The *para-*trifluoromethylbenzyl (**63**) and *para*-pyridyl (**64**) analogues appeared to substantially retain potency compared with TDR32750, although the straight benzyl derivative (**62**) showed around a fivefold loss in activity.

#### In vitro drug metabolism and pharmacokinetics (DMPK)

For key compounds, metabolic stability when incubated with mouse liver microsomes was measured, and for some compounds solubility was also assessed. For the A-ring variants, the solubility increased, particularly at low pH, probably due to the basic nature of the piperidine ring. In those derivatives with an aromatic or benzyl substituent, the basicity of the amine is decreased, which probably explains the lower solubility at pH 6.5.

Disappointingly, the in vitro intrinsic clearance (CL_int_) values were all rather high—the preferred value is <50 μL min^−1^ mg^−1^ protein, although the aim is for a value of less than 20 μL min^−1^ mg^−1^. The most stable compounds were **17**, **20**, **31**, **35, 40**, **48**, and **50**, with derivatives **20** and **31** being relatively inactive. Compound **48** is particularly stable, presumably due to the relatively low *c*log D value and the metabolic stability of the isoxazole ring.

For C-ring variants, the most active compounds (**56**–**58**) were investigated for microsomal stability. Compound **56** showed moderate stability, whilst the *N*-alkyl congeners were less stable, possibly due to CYP450-mediated metabolism of the alkyl groups.

### In vivo studies

Three compounds were selected for further study in vivo. Compound **43** was selected from the A-ring-modified series, as it represents a compound with good potency against the malaria parasite and very good selectivity compared with mammalian cells, albeit with a slightly increased microsomal instability. Compounds **56** and **57** were chosen as C-ring-modified derivatives, and also for their potency, selectivity and *c*log D value. Compounds **57** and **58** have very similar properties; compound **57** was chosen as it has marginally better properties. Multidose oral efficacy studies of compounds **43**, **56**, and **57** against green fluorescent protein (GFP)-transfected *P. berghei* ANKA-infected mice were conducted according to the standard protocol (Peters), with test compounds administered either per oral (po) or via intraperitoneal (ip) injection (Table 3).

**Table 3:** In vivo antimalarial activity against green fluorescent protein (GFP)-transfected *Plasmodium berghei* ANKA.[a]

Compd	Dose [mg kg^−1^ day^−1^]	Route	[%] Reduction parasitaemia	Survival [days]
**43**	50	ip	10.0	4
**56**	50	ip	21.8	4
**57**	50	ip	99.9	13.7
				
**43**	100	po	0.0	4
**56**	100	po	13.6	4
**57**	100	po	50.5	8
				
Chloroquine	10	ip	99.97	20
TDR32570	100	po	25.5	7.7
Untreated control	–	–	–	7

[a] Animals were dosed four times a day at the stated dose. Route of administration: per oral (po); intraperitoneal (ip). Formulation: 10 % DMSO in water.

Both via po and ip administration, compound **57** was the most efficacious in terms of decrease of parasitaemia. At a dose of 50 mg kg^−1^ ip, compound **57** decreased the level of parasitaemia by 99.9 %, and there was a significant increase in survival time. When dosed orally, the decrease in parasitaemia was less at 50.5 %. Compound **57** shows limited microsomal stability, so the decrease in oral activity of the compound is likely to be due to poorer exposure as a consequence of first-pass metabolism, but further work is required to confirm this hypothesis. Compound **43** failed to show significant activity in vivo, despite showing good potency in vitro. This might be due to poor pharmacokinetics, but this needs to be further investigated.

## Conclusions

Modifications to our prototypical lead compound TDR32750 have been shown to yield compounds that retain in vitro activity. These changes include removal of the ester functionality that might be a point of metabolism and addition of a basic centre that should be helpful in increasing the solubility. Although these changes have not yet provided compounds with good oral in vivo activity, the results do suggest that there is further scope for the optimisation of the metabolic and physicochemical properties of this series, which could potentially lead to orally active antimalarial compounds.

## Experimental Section

**Profiling software**: StarDrop version 5.3 with the P450 metabolism plug-in module (http://www.optibrium.com) was used to predict the sites of metabolism of the compounds. The same software was used to calculate the *c*Log D values for all compounds.

**Chemistry**: Chemicals and solvents were purchased from Sigma–Aldrich or Fluka and were used as received unless otherwise stated. Air-and moisture-sensitive reactions were carried out under an inert atmosphere of argon in oven-dried glassware. Analytical thin-layer chromatography (TLC) was performed on precoated TLC plates (layer 0.20 mm silica gel 60 with fluorescent indicator UV254; Merck). Developed plates were air-dried and analysed under a UV lamp (254/365 nm). Flash column chromatography was performed using prepacked silica gel cartridges (230–400 mesh, 40–63 μm; SiliCycle) using a Teledyne ISCO Combiflash Companion or Combiflash Retrieve. Microwave irradiation was conducted using a Biotage Initiator unit. The machine consists of a continuous focused microwave power delivery system with operator-selectable power output (0–400 W at 2.45 GHz). ^1^H NMR and ^13^C NMR spectra were recorded on a Bruker Avance II 500 spectrometer (^1^H at 500.1 MHz; ^13^C at 125.8 MHz) or a Bruker DPX300 spectrometer (^1^H at 300.1 MHz). Chemical shifts (*δ*) are expressed in ppm recorded using the residual solvent as the internal reference in all cases. Signal splitting patterns are described as singlet (s), doublet (d), triplet (t), quartet (q), pentet (p), multiplet (m), broad (br), or a combination thereof. Coupling constants (*J*) are quoted to the nearest 0.1 Hz. LCMS analyses were performed with either an Agilent HPLC 1100 series connected to a Bruker Daltonics MicroTOF or an Agilent Technologies 1200 series HPLC connected to an Agilent Technologies 6130 quadrupole spectrometer, where both instruments were connected to an Agilent diode array detector. Liquid chromatography–mass spectrometry (LCMS chromatographic separations were conducted with a Waters X bridge C18 column (50 mm×2.1 mm, 3.5 *μm* particle size), with a mobile phase of water/acetonitrile+0.1 % HCOOH, or water/acetonitrile+0.1 % NH_3_, using a linear gradient from 80:20 to 5:95 over 3.5 min and then held for 1.5 min, at a flow rate of 0.5 mL min^−1^. All compound samples evaluated in biological assays had a measured purity of ≥95 % (by total ion current (TIC) and UV) as determined using this analytical LCMS system. High-resolution mass spectrometry (HRMS) using electrospray ionisation was performed on a Bruker Daltonics MicrOTOF mass spectrometer.

**1-Benzyl-4-(2,5-dimethyl-1*H*-pyrrol-1-yl)piperidine (6)**: 2,5-Hexandione (**4**) (2.0 g, 17.5 mmol), 4-amino-1-benzylpiperidine (**5**) (4.0 g, 21.0 mmol) and *para*-toluenesulfonic acid (*p*-TsOH) bound to silica gel (0.4 equiv mol^−1^) were mixed in an oven-dried pressure vials with magnetic stir bars, and heated twice (180 °C, 15 min) under microwave irradiation (0–400 W at 2.45 GHz) and then stirred for 15 min at RT. The reaction mixture was filtered, and the silica gel residue was washed with CH_2_Cl_2_ (10 mL). The solvent was removed in vacuo to give compound **6** as a light brown oil (3.8 g, 90 %): ^1^H NMR (500 MHz, CDCl_3_): *δ*=7.43–7.33 (m, 5 H), 5.82 (s, 2 H), 4.01–3.94 (m, 1 H), 3.64 (s, 2 H), 3.11–3.09 (m, 2 H), 2.39 (br s, 6 H), 2.38–2.32 (m, 2 H), 2.19–2.12 (dt, 2 H, *J*=11.9 Hz), 1.89–1.85 ppm (m, 2 H); MS (ESI+): *m*/*z* (%): 269.4 [*M*+H]^+^ (100).

**1-(1-Benzylpiperidin-4-yl)-2,5-dimethyl-1*H*-pyrrole-3-carbaldehyde (7)**: General procedure A (below) was used to give desired product **7** as a dark brown oil (3.5 g, 92 %): ^1^H NMR (500 MHz, CDCl_3_): *δ*=9.78 (s, 1 H, C*H*O), 7.36–7.25 (m, 5 H), 6.25 (s, 1 H), 4.00–3.93 (m, 1 H), 3.56 (m, 2 H), 3.08–3.01 (m, 2 H), 2.58 (s, 3 H), 2.39–2.33 (m, 2 H), 2.31 (s, 3 H), 2.13–2.08 (dt, 2 H, *J*=11.8 Hz), 1.80–1.77 ppm (m, 2 H); MS (ESI+): *m*/*z* (%): 297.9 [*M*+H]^+^ (100).

***tert*****-Butyl 4-(2,5-dimethyl-1*H*-pyrrol-1-yl)piperidine-1-carboxylate (9)**: The procedure for **6** (see above) was used to give compound **9** as a brown oil (3.0 g, 62 %): ^1^H NMR (500 MHz, CDCl_3_): *δ*=5.68 (s, 2 H), 4.20 (br s, 2 H), 3.96 (tt, 1 H, *J*=7.5, 4.5 Hz), 2.67 (t, 2 H, *J*=7.5 Hz), 2.21 (s, 6 H), 2.04 (td, 2 H, *J*=12.4 Hz), 1.74 (d, 2 H, *J*=13.5 Hz), 1.41 ppm (br s, 9 H); MS (ESI+): *m*/*z* (%): 279.4 [*M*+H]^+^ (100).

**4-(2,5-Dimethylpyrrol-1-yl)piperidine (10)**: A solution of **9** (3.0 g, 0.010 mol) in 4 m HCl/dioxane (30 mL) was stirred at 0 °C for 3 h. Once the reaction was complete, the solvent was removed in vacuo. The residue was redissolved in CH_2_Cl_2_ (30 mL), washed with 10 % aq NaOH (30 mL), dried over MgSO_4_, filtered and concentrated in vacuo to afford product **10** as a colourless oil (1.8 g, 94 %): ^1^H NMR (500 MHz, CD_3_OD): *δ*=5.79 (s, 2 H), 3.94–3.88 (m, 1 H), 3.17–3.13 (m, 2 H), 2.66–2.60 (m, 2 H), 2.23 (s, 6 H), 2.10–2.02 (m, 2 H), 1.77–1.74 ppm (m, 2 H); MS (ESI+): *m*/*z* (%): 179.5 [*M*+H]^+^ (100).

**Alternate method for the synthesis of 4-(2,5-Dimethylpyrrol-1-yl)piperidine (10)**: A solution of 1-benzyl-4-(2,5-dimethyl-1*H*-pyrrol-1-yl)piperidine (**6**) (3.8 g, 0.014 mol) in MeOH (10 mL) was treated with a catalytic amount of Pd(OH)_2_. The mixture was stirred under a hydrogen atmosphere at RT overnight. Upon completion, the reaction mixture was filtered through Celite, and the filtrate was concentrated in vacuo to afford product **10** as a colourless oil, which was used without further purification (2.4 g, 96 %); ^1^H NMR (500 MHz, CD_3_OD): *δ*=5.79 (s, 2 H), 3.94–3.88 (m, 1 H), 3.17–3.13 (m, 2 H), 2.66–2.60 (m, 2 H), 2.23 (s, 6 H), 2.10–2.02 (m, 2 H), 1.77–1.74 ppm (m, 2 H); MS (ESI+): *m*/*z* (%): 179.5 [*M*+H]^+^ (100).

**General procedure A for the preparation of 2,5-dimethyl-1-(1-substituted-piperidine)-3-formylpyrroles** (**16 a–z, 16 aa–aj**): POCl_3_ (6 mmol, 6 equiv) was added dropwise to ice-cooled DMF (12 mL) under a N_2_ atmosphere. The mixture was allowed to warm to RT over 15 min, then a solution of the appropriate 2,5-dimethyl-1-(1-substituted-piperidine)-1*H*-pyrrole (**11 a**, **12 a**–**s**, **13 a**-**i**, **14 a**–**e**, **15**) (1 mmol, 1 equiv) in DMF (5 mL) was added, and the mixture was heated at 100 °C for 3 h. After cooling, 30 % aq NaOH was added dropwise to adjust the solution to approximately pH 10. The resulting precipitate was isolated by filtration, and washed with water, and dried in vacuo to afford the desired 2,5-dimethyl-1-aryl/substituted-aryl-3-formylpyrrole (**16 a**–**z**, **16 aa**–**aj**) (80–95 % yield).

**General procedure B for the preparation of (*E*)-ethyl 5-((1-substituted-piperidin-4-yl)-2,5-dimethyl-1*H*-pyrrol-3-yl)methylene)-2-methyl-4-oxo-4,5-dihydro-1*H*-pyrrole-3-carboxylates (17–54)**: A solution of **3** (1.0 molar equiv) in abs EtOH (3 mL) was treated with the appropriate 2,5-dimethyl-1-(1-substituted-piperidine)-3-formylpyrrole (**16 a**–**z**, **16 aa**–**aj**) (1.0 molar equiv) and KHSO_4_ (0.2 molar equiv). The reaction mixture was heated at 70–80 °C for 3 h and then poured onto crushed ice and filtered to afford the desired product (**17**–**54**) as a yellow powder (80–95 % yield).

**(*E*)-Ethyl 5-((1-(1-benzylpiperidin-4-yl)-2,5-dimethyl-1*H*-pyrrol-3-yl)methylene)-2-methyl-4-oxo-4,5-dihydro-1*H*-pyrrole-3-carboxylate (17)**: Yellow powder (0.100 g, 40 %): mp: 150–155 °C; ^1^H NMR (500 MHz, CD_3_OD): *δ*=10.13 (s, 1 H, N*H*), 7.36–7.30 (m, 5 H), 6.65 (s, 1 H), 6.51 (s, 1 H), 4.12–4.07 (m, 3 H), 3.55 (br s, 2 H), 2.95 (d, 2 H, *J*=7.0 Hz), 2.53 (s, 3 H), 2.43 (t, 2 H, *J*=5.7 Hz), 2.36 (s, 3 H), 2.32 (s, 3 H), 2.16 (d, 2 H, *J*=9.1 Hz), 1.75 (d, 2 H, *J*=10.4, Hz), 1.21 ppm (t, 3 H, *J*=7.1 Hz); ^13^C NMR (125 MHz, [D_6_]DMSO): *δ*=180.3, 178.3, 169.2, 164.2, 163.7, 163.3, 134.6, 130.1, 128.8, 128.5, 128.1, 126.9, 117.4, 113.0, 110.1, 102.4, 64.0, 61.7, 58.1, 57.8, 55.1, 54.6, 52.6, 30.5, 15.7, 14.4, 11.0 ppm; MS (ESI+): *m*/*z* (%): 448.3 [*M*+H]^+^ (100); HRMS–ESI: *m*/*z* [*M*+H]^+^ calcd for C_28_H_34_N_3_O_3_: 448.2469, found: 448.2468.

**(*E*)-Ethyl 5-((2,5-dimethyl-1-(2-(trifluoromethyl)phenyl)-1*H*-pyrrol-3-yl)methylene)-2-((*E*)-2-(dimethylamino)vinyl)-4-oxo-4,5-dihydro-1*H*-pyrrole-3-carboxylate (55)**: A mixture of TDR32750 (0.02 g, 0.004 mmol) and DMF–diethyl acetal (3 mL) was heated at reflux for 15 min. After cooling, the precipitate was isolated by filtration, washed with ice-cold water, and dried in vacuo to afford desired enamino ester **55** as a yellow powder (0.22 g, 89 %): mp: 270–275 °C; ^1^H NMR (500 MHz, [D_6_]DMSO): *δ*=9.05 (s, 1 H, N*H*), 8.14 (d, 1 H, *J*=13.3 Hz), 8.00 (d, 1 H, *J*=7.4 Hz), 7.91 (t, 1 H, *J*=7.4 Hz), 7.81 (t, 1 H, *J*=7.7 Hz), 7.47 (d, 1 H, *J*=7.8 Hz), 6.64 (s, 1 H), 6.32 (s, 1 H), 6.09 (d, 1 H, *J*=13.3 Hz), 4.13–4.07 (m, 2 H), 2.51 (q, 6 H, *J*=7.1 Hz), 1.95 (s, 3 H), 1.91 (s, 3 H), 1.23–121 ppm (m, 3 H, *J*=2.4, 7.05 Hz); ^13^C NMR (125 MHz, [D_6_]DMSO): *δ*=179.9, 170.2, 165.7, 164.2, 163.2, 151.7, 134.2, 132.4, 131.8, 130.2, 114.2, 113.5, 109.3, 106.4, 105.9, 102.4, 102.3, 96.8, 84.8, 57.6, 15.8, 14.5, 14.4, 12.0, 10.2 ppm; HRMS–ESI: *m*/*z* [*M*+H]^+^ calcd for C_25_H_27_F_3_N_3_O_3_: 474.1999, found: 474.2002.

**General procedure for the preparation of pyrrolones 56**–**64**: A mixture of enamino ester **55** (1.0 equiv) and the appropriate primary amine (4.0 equiv) in *iso*-propanol was heated at reflux for 6 h or irradiated by microwave (0–400 W at 2.45 GHz) at 110 °C for 15–30 min and then cooled. The resultant precipitate was isolated by filtration, washed with ice-cold MeOH or EtOH, and dried in vacuo to afford the desired product (**56**–**64**).

**(*E*)-5-Benzyl-2-((2,5-dimethyl-1-(2-(trifluoromethyl)phenyl)-1*H*-pyrrol-3-yl)methylene)-1*H*-pyrrolo[3,2-*c*]pyridine-3,4(2*H*, 5*H*)dione (62)**: Isolated as a yellow powder (0.22 g, 89 %): mp: 195–200 °C; ^1^H NMR (500 MHz, [D_6_]DMSO): *δ*=9.45 (s, 1 H, N*H*), 8.00 (d, 1 H, *J*=7.8 Hz), 7.93 (t, 2 H, *J*=7.6 Hz), 7.83 (t, 1 H, *J*=7.2 Hz), 7.68 (d, 2 H, *J*=8.2 Hz), 7.59 (d, 2 H, *J*=8.2 Hz), 7.49 (d, 1 H, *J*=7.8 Hz), 6.83 (br s, 2 H), 6.54 (s, 1 H), 6.38 (d, 1 H, *J*=7.2 Hz), 5.27–5.26 (m, 2 H), 1.82 ppm (s, 6 H); ^13^C NMR (125 MHz, [D_4_]acetone): *δ*=181.7, 170.8, 162.3, 161.0, 158.2, 145.2, 143.6, 136.0, 134.8, 132.6, 132.3 (2C), 131.0 (2C), 130.8, 130.3, 129.3, 129.2, 128.3, 126.3, 126.2, 115.6, 108.7, 106.5, 95.6, 50.6, 12.3, 10.7 ppm; MS (ESI+): *m*/*z* (%): 490.18 [*M*+H]^+^ (100); HRMS–ESI: *m*/*z* [*M*+H]^+^ calcd for C_28_H_23_F_3_N_3_O_2_: 490.1737, found: 490.1752.

**In vivo efficacy studies**: The protocols are described in the Supporting Information. In vivo efficacy studies in mice were conducted at the Swiss Tropical & Public Health Institute (Basel, Switzerland) according to the rules and regulations for the protection of animal rights (“Tierschutzverordnung”) of the Swiss “Bundesamt für Veterinärwesen”. They were approved by the veterinary office of Canton Basel-Stadt, Switzerland.

### Supporting Information

Supporting Information contains: synthetic routes for the synthesis of intermediates and characterisation data for compounds not included in the main text; methods for physicochemical evaluation of the compounds and the data generated; methods for assessing metabolic stability; in vitro and in vivo parasite testing.

This material is available free of charge on the WWW under http://dx.doi.org/10.1002/cmdc.201300177.

## Notes

Some of the compounds had a trace, inseparable amount of (*Z*)-isomer. This could not be detected in the ^13^C NMR spectra and was only detected using UPLC.
