# Supplementary material for: Structure–Activity Relationship Studies of Pyrrolone Antimalarial Agents
Source: ChemMedChem. 2013 Aug 5;8(9):1537–44. doi: 10.1002/cmdc.201300177 (PMC3963473; doi:10.1002/cmdc.201300177)
Supplement: Supplementary file 1 [file cmdc0008-1537-sd1.pdf]

CHEMMEDCHEM
Chemistry & Drug Discovery

Supporting Information

© Copyright Wiley-VCH Verlag GmbH & Co. KGaA, 69451 Weinheim, 2013

Structure–Activity Relationship Studies of Pyrrolone Antimalarial Agents

Dinakaran Murugesan,[a] Marcel Kaiser,[b] Karen L. White,[c] Suzanne Norval,[a] Jennifer Riley,[a] Paul G. Wyatt,[a] Susan A. Charman,[c] Kevin D. Read,[a] Clive Yeates,[d] and Ian H. Gilbert*[a]

cmdc_201300177_sm_miscellaneous_information.pdf

Supporting Information

Table of contents

I.	General information	Page S2
II.	Synthesis and Spectral data	Page S3
III.	Physicochemical Evaluation	Page S49
IV.	Metabolism and Efficacy	Page S50
V.	Design and Results of the in vitro & in vivo Studies	Page S51
VI.	Additional References	Page S53

I. General information

^{1}H NMR and ^{13}C NMR spectra were recorded on a Bruker Avance II 500 spectrometer (^{1}H at 500.1 MHz, ^{13}C at 125.8 MHz) or a Bruker DPX300 spectrometer (1H at 300.1 MHz). Chemical shifts (*δ*) are expressed in ppm recorded using the residual solvent as the internal reference in all cases. Signal splitting patterns are described as singlet (s), doublet (d), triplet (t), quartet (q), pentet (p), multiplet (m), broad (br), or a combination thereof. Coupling constants (*J*) are quoted to the nearest 0.1 Hz. LC−MS analyses were performed with either an Agilent HPLC 1100 series connected to a Bruker Daltonics MicroTOF or an Agilent Technologies 1200 series HPLC connected to an Agilent Technologies 6130 quadrupole spectrometer, where both instruments were connected to an Agilent diode array detector. LC−MS chromatographic separations were conducted with a Waters Xbridge C18 column, 50 mm × 2.1 mm, 3.5 *μ*m particle size; mobile phase, water/acetonitrile + 0.1% HCOOH, or water/acetonitrile + 0.1% NH_3; linear gradient from 80:20 to 5:95 over 3.5 min and then held for 1.5 min; flow rate of 0.5 mL min^{-1}. All assay compounds had a measured purity of ≥95% (by TLC and UV) as determined using this analytical LC−MS system. High resolution electrospray measurements were performed on a Bruker Daltonics MicroTOF mass spectrometer.

II. Synthesis and Spectral Data:

Synthesis of ethyl (E)-3-amino-2-(2-chloroacetyl)but-2-enoate (2).

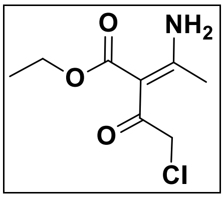

A solution of ethyl-3-aminocrotonate (2.0 g, 0.015 mol, 1.0 equiv) and pyridine (1.2 g, 0.015 mol, 1.0 equiv) in diethylether (10 mL) was cooled to 0 ºC. Chloroacetylchloride (4.1 g, 0.037 mol, 2.4 equiv) dissolved in diethylether (5 mL) was added drop wise during 30 min, maintaining the temperature 0 ºC. The reaction mixture was stirred for 3 h at 0 ºC and the solvent then removed *in vacuo*. The resultant solid was washed with cold water to yield the desired product **2,** as a cream yellow powder (2.7 g, 87%), mp 131-132 ºC. ^{1}H NMR (500 MHz, DMSO-d_6): *δ* ppm. 5.92 (br s, 2H, NH_2), 4.57 (s, 2H, -CH_2Cl), 4.27 (q, 2H,-OCH_2, *J* = 7.2 Hz), 2.36 (s, 3H, CH_3), 1.36 (t, 3H, OCH$_2$CH_3, *J* = 7.2 Hz). ^{13}C NMR (125 MHz, DMSO-d_6): *δ* ppm 190.6, 169.9, 168.3, 100.8, 60.5, 49.6, 24.6, 14.3. MS (ESI$^+$): m/z 206.11 [M+1]$^+$ 100%.

Synthesis of ethyl 5-methyl-3-oxo-1,2-dihydropyrrole-4-carboxylate (3).

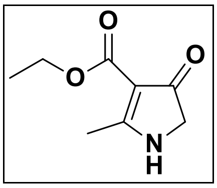

Ethyl (E)-3-amino-2-(2-chloroacetyl)but-2-enoate **(2)** (2.0 g, 0.0097 mol, 1.0 equiv) was dissolved in absolute ethanol (5 mL) and cooled to 0 ºC. Potassium hydroxide (1.09 g, 0.019 mol, 20 equiv) was added and stirred for 3 h at 0 ºC. After completion of reaction, the solution was acidified to pH 2.0 using 2N HCl to afford a yellow precipitate, which was washed with cold water to yield the required product **3** as a yellow solid (1.6 g, 99%), mp 215 ºC. ^{1}H NMR (500 MHz, DMSO-d_6): (Keto-Enol form) *δ* ppm. 10.7 (br s, 1H, -O*H*), 9.4 (br s, 1H,-N*H*), 7.64 (s, 1H, -N*H*), 6.05 (d, 1H, *J* = 2.4 Hz), 4.27 (q, 2H,-OCH_2, *J* = 7.1 Hz), 4.08 (m, 2H, -OCH_2), 3.80 (d, 2H), 2.4 (t, 3H, *J* = 1.6 Hz), 2.3 (s, 3H), 1.8 (t, 3H, *J* = 7.1 Hz), 1.2 (t, 3H, *J* = 7.0 Hz, OCH$_2$CH_3). ^{13}C NMR (125 MHz, DMSO-d_6): *δ* ppm 189.2, 171.5, 159.9, 99.3, 86.1, 60.4, 28.02, 14.2. MS (ESI$^+$): m/z 170.14 [M+1]$^+$ 100%.

A. General procedure for the microwave-accelerated synthesis of 2,5-dimethyl-1-aryl-1*H*-pyrroles.

2,5-Hexandione **(4)** (1 mmol), the appropriate amines **(5** or **8)** (1.2 equiv) and *p*-toluenesulfonic acid bound to silica gel (0.4 equiv) were mixed in oven dried pressure vials with magnetic stir bars and heated twice (180 ºC, 5 min) under microwave irradiation (0-400 W at 2.45 GHz). After reaction the vessels were stirred for 15 min at room temperature. The reaction mixtures were filtered and the silica removed and washed with DCM (10 mL). The solvent was removed under reduced pressure affording the desired 2,5-dimethyl-1-substituted-1*H*-pyrrole **(6** or **9)** (Purity >95%, 80-90% yield).

B. Prototypical procedure for reductive amination: 4-(2,5-dimethyl-1*H*-pyrrol-1-yl)-1-(2-(trifluoromethyl)benzyl)piperidine (12e).

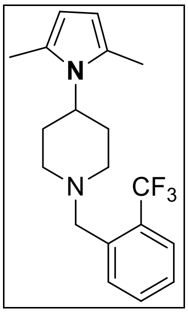

To a solution of 4-(2,5-dimethyl-1*H*-pyrrol-1-yl)piperidine (**10)** (0.200 g, 1.12 mmol) and 2-(trifluoromethyl)benzaldehyde (0.195 g, 1.12 mmol) in acetonitrile (5 mL) was added sodium triacetoxyborohydride (0.475 g, 2.26 mmol) and the reaction stirred for 8 h, at r.t. Once the reaction was completed, saturated sodium bicarbonate solution (20 mL) was added and the mixture stirred for 10 min, extracted with ethylacetate (15 mL x 2), dried over magnesium sulphate and concentrated to afford **12e** as a oil (0.270 g, 73%). ^{1}H NMR (500 MHz, $CDCl_3$): δ ppm. 7.80–7.78 (d, 1H, *J* = 7.7 Hz), 7.61–7.60 (d, 1H, *J* = 7.7 Hz), 7.54–7.51 (t, 1H, *J* = 7.5, 7.6 Hz), 7.34–7.31 (t, 1H, *J* = 7.6, 7.5 Hz), 6.59 (s, 2H), 4.04–3.97 (m, 1H), 3.68 (s, 2H), 3.00–2.98 (d, 2H, J=2.0, 7.7 Hz), 2.35-2.32 (m, 1H), 2.31 (s, 6H), 2.21–2.16 (m, 2H), 2.13 (s, 1H), 1.79–1.77 (m, 2H). MS (ESI^+): m/z 337.14 $[M+1]^+$ 100%.

C. Prototypical procedure for alkyl/arylation: 3-((4-(2,5-dimethyl-1*H*-pyrrol-1-yl)piperidin-1-yl)methyl)pyridine (13a).

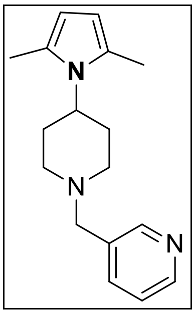

To a solution of 4-(2,5-dimethyl-1*H*-pyrrol-1-yl)piperidine (**10**) (0.102 g, 0.54 mmol) and 3-(bromomethyl)pyridine (0.144 g, 0.5 mmol) dissolved in DMF, was added DIPEA (0.147 g, 1.1 mol) and the reaction mixture was heated at reflux for 3h at 70 °C. Once the reaction was completed, the mixture was extracted with dichloromethane/water. The organic layer was dried over magnesium sulphate and concentrated to afford 3-((4-(2,5-dimethyl-1*H*-pyrrol-1-yl)piperidin-1-yl)methyl)pyridine (**13a**) as a light brown oil (0.142 g, 92%). ^{1}H NMR (500 MHz, $CDCl_3$): *δ* ppm. 8.58 (d, 1H, *J* = 1.5 Hz), 8.55–8.54 (dd, 1H, *J* = 4.6, 4.8 Hz), 7.71–7.69 (dt, 1H, *J* = 7.7, 7.8 Hz), 7.31–7.30 (td, 1H, *J* = , 4.7, 7.8, Hz), 5.54 (s, 2H), 4.02–3.96 (tt, 1H, *J* = 12.5 Hz), 3.55–3.58 (d, 2H, *J* = 3.7 Hz), 3.05–3.03 (dq, 2H, *J* = 9.6 Hz), 2.40–2.34 (m, 2H), 2.32 (s, 6H), 2.18–2.12 (td, 2H, *J* = 11.7, 11.8 Hz), 1.84–1.81 (dq, 2H, *J* = 11.6, 12.1 Hz). MS (ESI$^+$): m/z 270.22 $[M+1]^+$ 100%.

D. Prototypical procedure for coupling: 5-((4-(2,5-Dimethyl-1*H*-pyrrol-1-yl)piperidin-1-yl)methyl)-2-(trifluoromethyl)pyridine (14e).

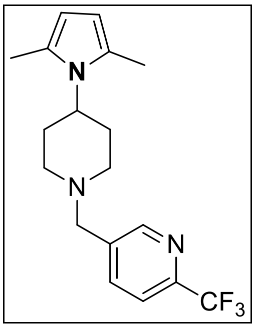

(6-(Trifluoromethyl)pyridin-3-yl)methanol (0.200 g, 0.00112 mol) was dissolved in dichloromethane (5 mL) and cooled to 0 °C. Methanesulfonyl chloride (0.129 g, 1.2 mmol) was added slowly, followed by triethylamine (0.157 mL, 1.12 mmol). After 1 h, the suspension was poured into ice-cold water. The aqueous phase was extracted with

dichloromethane, and the combined organic extracts were dried over magnesium sulphate, filtered and evaporated to give the mesylate as a white oil (0.3 g, 99%) which was used without further purification. The mesylate (0.3 g, 1.5 mmol), 4-(2,5-dimethyl-1*H*-pyrrol-1-yl)piperidine (**10**) (0.271 g, 0.15 mmol) and triethylamine (0.4 mL, 3.0 mmol) were dissolved in acetonitrile (3 mL).and $NaHCO_3$ (0.2 mg, 0.003 mmol) added. The suspension was stirred and heated at reflux for 12 h. After cooling to room temperature the solvent was evaporated, and the residue was partitioned between water and ethyl acetate. The organic phase was separated and dried over magnesium sulphate, filtered, and evaporated to dryness. The residue was subjected to flash chromatography on silica gel to give **14e** as colourless oil (0.250 g, 63%). ^{1}H NMR (500 MHz, $CDCl_3$): *δ* ppm. 8.55 (d, 1H), 7.78–7.76 (dd, 1H, *J* = 6.5 Hz), 7.54–7.52 (d, 1H, *J* = 7.8 Hz), 5.61 (s, 2H), 3.85–3.79 (tt, 1H, *J* = 4.0, 4.2 Hz), 3.50 (br s, 2H), 2.87–2.85 (m, 2H), 2.18 (s, 6H), 2.17–2.13 (d, 1H, *J* = 0.4 Hz), 1.90 (s, 1H), 2.08–2.03 (m, 2H), 1.70–1.67 (m, 2H). MS (ESI$^+$): m/z 338.15 [M+1]$^+$ 100%.

4-(2,5-Dimethyl-1*H*-pyrrol-1-yl)-1-methylpiperidine (11a).

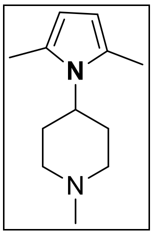

General Procedure A to give (**11a**) as a brown oil (1.68 g, 54%). ^{1}H NMR (500 MHz, $CDCl_3$): *δ* ppm. 5.74 (s, 2H), 3.91–3.86 (tt, 1H, *J* = 8.5, 4.0 Hz), 3.01-2.98 (d, 2H, *J* = 11.4 Hz), 2.36–2.33 (dd, 2H, *J* = 12.5 Hz), 2.30 (s, 3H), 2.19 (s, 6H), 2.01–2.06 (td, 2H, *J* = 11.4 Hz), 1.83–1.79 (d, 2H, *J* = 12.4 Hz). MS (ESI$^+$): m/z 193.22 [M+1]$^+$ 100%.

4-(2,5-Dimethyl-1*H*-pyrrol-1-yl)-1-propylpiperidine (12a).

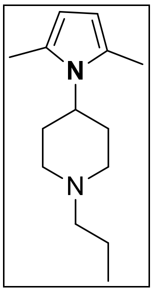

General Procedure B to give (**12a**) as a brown oil (0.25 g, 90%). ^{1}H NMR (500 MHz, $CDCl_3$): *δ* ppm. 6.35 (s, 2H), 3.96–3.89 (m, 1H), 3.06–3.04 (d, 2H, *J* = 2.1 Hz), 2.91 (s,1H), 2.53 (s, 6H), 2.32–2.30 (m, 2H), 2.29 (s, 1H), 2.26 (s, 3H), 2.04–1.99 (td, 1H, *J* = 11.9, 2.2 Hz), 1.78–1.75 (m, 2H), 0.89–0.86 (t, 3H, *J* = 7.3, 7.5 Hz). MS (ESI$^+$): m/z 221.14 $[M+1]^+$ 100%.

4-(2,5-Dimethyl-1*H*-pyrrol-1-yl)-1-isopentylpiperidine (12b).

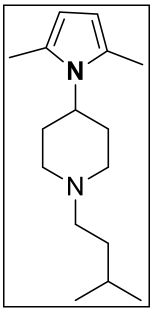

General Procedure B to give (**12b**) as a brown oil (0.027 g, 99%). ^{1}H NMR (500 MHz, $CDCl_3$): *δ* ppm. 5.68 (s, 2H), 3.93–3.88 (t, 1H, *J* = 12.2 Hz), 3.06 (br s, 2H), 2.51 (s, 3H), 2.34 (br s, 2H), 2.24 (s, 3H), 2.01 (br s, 2H), 1.77–1.72 (d, 2H, *J* = 12.1 Hz), 1.57–1.49 (m, 3H), 1.36(br s, 2H), 0.92–0.91 (d, 3H, *J* = 6.7 Hz), 0.85–0.84 (d, 3H, *J* = 6.6 Hz). MS (ESI$^+$): m/z 249.14 $[M+1]^+$ 100%.

4-(2,5-Dimethyl-1*H*-pyrrol-1-yl)-1-neopentylpiperidine (12c).

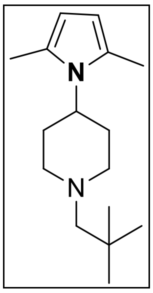

General Procedure B to give (**12c**) as a brown oil (0.13 g, 95%). ^{1}H NMR (500 MHz, $CDCl_3$): *δ* ppm. 6.29 (s, 2H), 3.86 -3.81 (tt, 1H, *J* = 4.2, 4.0 Hz), 2.77 (s, 2H), 2.30–2.25 (td, 2H, *J* = 11.7, 10.7 Hz), 2.21 (s, 6H), 2.20–2.15 (dd, 2H, *J* = 12.0, 12.2 Hz), 2.00 (s, 2H), 1.63–1.60 (m, 2H), 0.78 (s, 9H). MS (ESI$^+$): m/z 249.20 $[M+1]^+$ 100%.

1-(Cyclopropylmethyl)-4-(2,5-dimethyl-1*H*-pyrrol-1-yl)piperidine (12f).

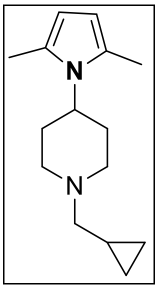

General Procedure B to give (**12f**) as a brown oil (0.20 g, 76%). ^{1}H NMR (500 MHz, $CDCl_3$): δ ppm. 6.29 (s, 2H), 3.84–3.82 (tt, 1H, *J* = 3.9 Hz), 3.14–3.12 (q, 2H, *J* = 2.1, 7.75 Hz), 2.84 (s, 6H), 2.47 (s, 3H), 2.27–2.21 (td, 1H, *J* = 3.7, 8.8 Hz), 2.02–1.97 (td, 2H, *J* = 10.00, 11.9 Hz), 1.73–1.70 (d, 2H, *J* = 1.4 Hz), 0.79–0.73 (m, 1H), 0.44-0.41 (m, 2H, *J* = 4.5Hz), 0.02–0.00 (m, 2H). MS (ESI$^+$): m/z 233.20 [M+1]$^+$ 100%.

1-(Cyclohexylmethyl)-4-(2, 5-dimethyl-1*H*-pyrrol-1-yl)piperidine (12g).

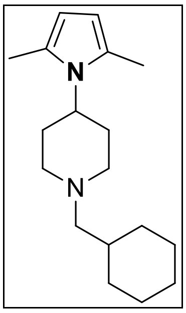

General Procedure B to give (**12g**) as a brown oil (0.250 g, 81%). ^{1}H NMR (500 MHz, $CDCl_3$): δ ppm. 5.70 (s, 2H), 3.86–3.79 (m, 1H), 2.89–2.87 (d, 2H, *J* = 10.2 Hz), 2.34 (s, 6H), 2.15–2.12 (m, 1H), 2.02–2.01 (d, 2H, *J* = 7.1 Hz), 1.90–1.85 (m, 2H), 1.66–1.64 (d, 4H, *J* = 1.4 Hz), 1.60–1.53 (m, 3H), 1.38–1.30 (m, 2H), 1.15–1.01 (m, 3H), 0.77–0.70 (m, 2H). MS (ESI$^+$): m/z 275.15 [M+1]$^+$ 100%.

4-(2,5-Dimethyl-1*H*-pyrrol-1-yl)-1-((1-methylpiperidin-4-yl)methyl)piperidine (12h).

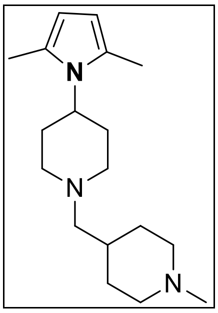

General Procedure B to give (**12h**) as a brown oil (0.148 g, 55%). ^{1}H NMR (500 MHz, $CDCl_3$): *δ* ppm. 6.57 (s, 2H), 3.92–3.86 (tt,1H, *J* = 3.8 Hz), 3.62 (br s, 1H), 3.36 (s, 2H), 2.96–2.94 (d, 2H, *J* = 9.7 Hz), 2.82–2.80 (m, 2H), 2.51 (s, 3H), 2.31 (s, 6H), 2.15–2.14 (d, 2H, *J* = 7.1 HZ), 1.99–1.93 (m, 2H), 1.90–1.85 (m, 2H), 1.71–1.69 (br s, 4H), 1.45–1.36 (m, 1H), 1.24–1.14 (m, 1H). MS (ESI$^+$): m/z 290.41 $[M+1]^+$ 100%.

4-(2,5-Dimethyl-1*H*-pyrrol-1-yl)-1-(4-fluoro-2-(trifluoromethyl)benzyl)piperidine (12i).

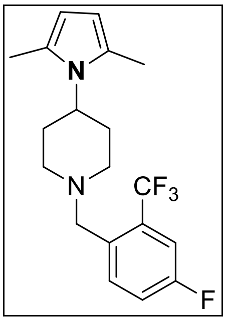

General Procedure B to give (**12i**) as a brown oil (0.156 g, 28%). ^{1}H NMR (500 MHz, $(CD_3)_2CO$): *δ* ppm. 7.98 (br s, 2H), 7.50–7.46 (m, 1H), 6.18 (s, 2H), 4.24–4.21 (tt, 1H, *J* = 3.7 Hz), 3.71 (s, 2H), 3.02–3.00 (d, 2H, *J* = 9.3 HZ), 2.61 (s, 2H), 2.39 (s, 6H), 2.32–2.27 (m, 2H), 1.86–1.84 (d, 2H, *J* = 3.6 Hz). MS (ESI$^+$): m/z 355.14 $[M+1]^+$ 100%.

4-(2,5-Dimethyl-1*H*-pyrrol-1-yl)-1-((1-methyl-1*H*-pyrazol-3-yl)methyl)piperidine (12j).

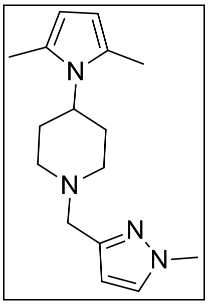

General Procedure B to give (**12j**) as a brown oil (0.150 g, 98%). ^{1}H NMR (500 MHz, $CDCl_3$): *δ* ppm. 7.22–7.21 (d, 1H, *J* = 2.1 Hz), 6.12 (d, 1H, *J* = 2.1 Hz), 5.64 (s, 2H), 4.11–4.05 (tt, 1H, *J* = 12.5 Hz), 3.79 (s, 3H), 3.02–2.99 (dq, 2H, *J* = 13.1 Hz), 2.28–2.25 (m, 2H), 2.26–2.23 (td, 2H, *J* = 12.8 Hz), 2.21 (s, 6H), 2.07–2.02 (td, 2H, *J* = 12.6 Hz), 1.74–1.69 (d, 2H, *J* = 13.1 Hz). MS (ESI^{+}): m/z 273.15 $[M+1]^{+}$ 100%.

1-((1*H*-Imidazol-2-yl)methyl)-4-(2,5-dimethyl-1*H*-pyrrol-1-yl)piperidine (12k).

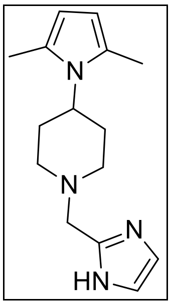

General Procedure B to give (**12k**) as a brown oil (0.250 g, 86%). ^{1}H NMR (500 MHz, $CDCl_3$): *δ* ppm. 7.28 (s, 2H), 7.05 (s, 2H), 4.04–3.95 (m, 1H), 3.73 (s, 2H), 3.05–3.02 (d, 2H, *J* = 10.3 Hz), 2.35 (s, 1H), 2.31 (s, 6H), 2.29–2.24 (m, 2H), 2.19 (s, 1H), 1.86–1.83 (m, 2H). MS (ESI^{+}): m/z 259.21 $[M+1]^{+}$ 100%.

3-((4-(2,5-Dimethyl-1*H*-pyrrol-1-yl)piperidin-1-yl)methyl)-5-methylisoxazole (12l).

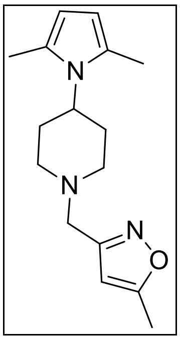

General Procedure B to give (**12l**) as a brown oil (0.200 g, 90%). ^{1}H NMR (500 MHz, $CDCl_3$): δ ppm. 5.54 (s, 2H), 6.04–6.03 (d, 1H, J = 0.7 Hz), 4.00–3.95 (m, 1H), 3.63 (s, 2H), 3.07–3.05 (m, 2H), 2.98 (s, 1H), 2.90 (d, 1H, J = 0.5 Hz), 2.59 (s, 3H), 2.37–2.34 (m, 1H), 2.30 (s, 6H), 2.24–2.22 (dd, 1H, J = 9.8 Hz), 1.84–1.81 (m, 1H), 1.73 (s, 1H). MS (ESI$^+$): m/z 274.15 [M+1]$^+$ 100%.

4-((4-(2,5-Dimethyl-1*H*-pyrrol-1-yl)piperidin-1-yl)methyl)oxazole (12m).

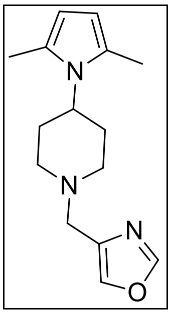

General Procedure B to give (**12m**) as a brown oil (0.240 g, 82%). ^{1}H NMR (500 MHz, $CDCl_3$): δ ppm. 7.99 (s, 1H), 7.87 (s, 1H), 5.77 (s, 2H), 3.97–3.91 (m, 1H), 3.56 (s, 2H), 3.12–3.07 (d, 2H, J = 1.9, 7.9 Hz), 2.55 (s, 2H), 2.31 (s, 6H), 2.20–2.15 (m, 2H), 1.81–1.78 (d, 2H, J = 1.7, 10.4 Hz). MS (ESI$^+$): m/z 260.15 [M+1]$^+$ 100%.

4-(2,5-Dimethyl-1*H*-pyrrol-1-yl)-1-((1-methyl-1*H*-imidazol-2-yl)methyl)piperidine (12n).

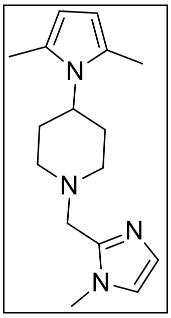

General Procedure B to give (**12n**) as a brown oil (0.280 g, 75%). ^{1}H NMR (500 MHz, $CDCl_3$): δ ppm. 6.84 (s, 1H), 6.76 (s, 1H), 5.63 (s, 2H), 4.03–4.01 (m, 1H, *J* = 7.1 Hz), 3.64 (s, 2H), 3.56 (br s, 1H), 2.93–2.91 (dd, 2H, *J* = 9.5 Hz), 2.49 (s, 3H), 2.21(s, 3H), 2.18–2.17 (d, 1H, *J* = 3.00 Hz), 2.15 (s, 1H), 2.13 (d, 1H, *J* = 1.53 Hz), 1.75–1.72(dd, 2H, *J* = 3.9 Hz), 1.18 (br s, 3H). MS (ESI^{+}): m/z 273.45 $[M+1]^{+}$ 100%.

4-(2,5-Dimethyl-1*H*-pyrrol-1-yl)-1-((1-methyl-1*H*-pyrrol-2-yl)methyl)piperidine (12o).

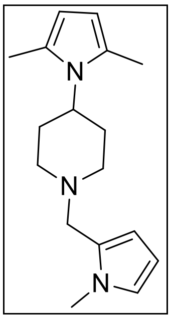

General Procedure B to give (**12o**) as a brown oil (0.220 g, 88%). ^{1}H NMR (500 MHz, $CDCl_3$): δ ppm. 6.47–6.46 (m, 1H), 5.92–5.90 (m, 1H), 5.88–5.87 (m, 1H), 5.61 (s, 2H), 3.84 (s, 1H), 3.54 (br s, 3H), 3.33 (br s, 2H), 2.89–2.87 (m, 2H), 2.17 (br s, 6H), 2.11–2.10 (d, 1H, *J* = .4 Hz), 2.08–2.07 (d, 1H, *J* = 4.3 Hz), 1.93–1.88 (m, 2H), 1.67–1.65 (m, 2H). MS (ESI^{+}): m/z 272.21 $[M+1]^{+}$ 100%.

3-(4-((4-(2,5-Dimethyl-1*H*-pyrrol-1-yl)piperidin-1-yl)methyl)phenyl)-5-methyl-1,2,4-oxadiazole (12p).

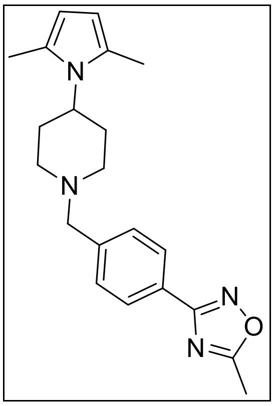

General Procedure B to give (**12p**) as a brown oil (0.150 g, 72%). ^{1}H NMR (500 MHz, $CDCl_3$): *δ* ppm. 7.94–7.93 (d, 2H, *J* = 8.2 Hz), 7.37–7.35 (d, 2H, *J* = 8.2 Hz), 5.65 (s, 2H), 3.85–3.78 (tt, 1H, *J* = 12.3 Hz), 3.51 (s, 2H), 2.95–2.92 (dq, 2H, *J* = 13.1 Hz), 2.55 (s, 3H), 2.21-2.17(m, 2H), 2.22 (s, 6H), 2.05–2.00 (td, 2H, *J* = 12.8 Hz), 1.72–1.69 (d, 2H, *J* = 13.1 Hz). MS (ESI$^+$): m/z 351.45 [M+1]$^+$ 100%.

4-(5-((4-(2,5-Dimethyl-1*H*-pyrrol-1-yl)piperidin-1-yl)methyl)pyridin-2-yl)morpholine (12q).

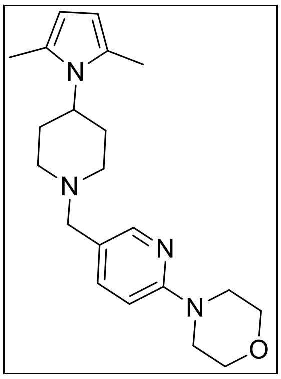

General Procedure B to give (**12q**) as a brown oil (0.210 g, 72%). ^{1}H NMR (500 MHz, $CDCl_3$): *δ* ppm. 8.13–8.12 (d, 1H, *J* = 2.0 Hz), 7.53–7.51 (dd, 1H, *J* = 2.1, 6.5 Hz), 6.68–6.65 (dd, 1H, *J* = 3.0, 5.6 Hz), 6.27 (s, 2H), 4.00–3.93 (m, 1H), 3.86–3.83 (m, 4H, *J* = 5.0 Hz), 3.53–3.51 (m, 5H), 3.47 (br s, 2H), 3.05–3.03 (d, 2H, *J* = 11.6 Hz), 2.59 (s, 3H), 2.31 (s, 3H), 2.30–2.26 (m, 1H), 2.19 (s, 1H), 2.11–2.07 (m, 2H), 1.76 (br s, 1H). MS (ESI$^+$): m/z 355.16 [M+1]$^+$ 100%.

4-(5-((4-(2,5-Dimethyl-1*H*-pyrrol-1-yl)piperidin-1-yl)methyl)pyrimidin-2-yl)morpholine (12r).

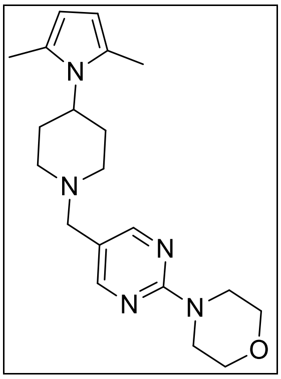

General Procedure B to give (**12r**) as a brown oil (0.180 g, 90%). ^{1}H NMR (500 MHz, $CDCl_3$): *δ* ppm. 8.20 (s, 2H), 7.19 (s, 2H), 3.92–3.85 (m,1H, *J* = 3.9 Hz), 3.73–3.70 (m, 7H), 3.33 (s, 2H), 2.97–2.94 (d, 2H, *J* = 11.4 Hz), 2.50 (s, 3H), 2.22 (s, 3H), 2.18 (s, 1H), 2.04–1.99 (d, 2H, *J* = 11.4 Hz), 1.97 (s, 1H), 1.74–1.71 (d, 2H, *J* = 1.7, 10.4 Hz). MS (ESI^+): m/z 356.30 $[M+1]^+$ 100%.

2-((4-(2,5-Dimethyl-1*H*-pyrrol-1-yl)piperidin-1-yl)methyl)pyridine (13b).

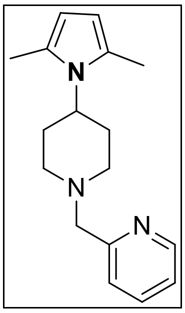

General Procedure C to give (**13b**) as a brown oil (0.150 g, 95%). ^{1}H NMR (500 MHz, $CDCl_3$): *δ* ppm. 8.58–8.56 (d, 1H, *J* = 1.5 Hz), 7.70-7.66 (tt, 1H, *J* = 7.6 Hz), 7.43-7.41 (d, 1H, *J* = 7.8 Hz), 7.20–7.18 (td, 1H, *J* = 7.8, Hz), 5.68 (s, 2H), 4.02-3.96 (tt, 1H, *J* = 12.5 Hz), 3.55-3.58 (d, 2H, *J* = 3.7 Hz), 3.05–3.03 (dq, 2H, *J* = 9.6 Hz), 2.40–2.34 (m, 2H), 2.32 (s, 6H), 2.18–2.12 (td, 2H, *J* = 11.7, 11.8 Hz), 1.84–1.81 (dq, 2H, *J* = 11.6, 12.1 Hz). MS (ESI^+): m/z 270.20 $[M+1]^+$ 100%.

4-(2,5-Dimethyl-1*H*-pyrrol-1-yl)-1-((tetrahydro-2*H*-pyran-4-yl)methyl)piperidine (13l).

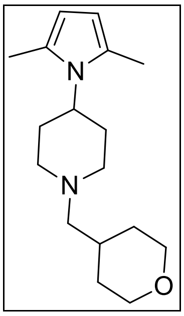

General Procedure C to give (**13l**) as a brown oil (0.156 g, 28%). ^{1}H NMR (500 MHz, $CDCl_3$): *δ* ppm. 5.64 (s, 2H), 3.91–3.84 (m, 4H), 3.34–3.29 (td, 2H, *J* = 11.9, 11.6 Hz), 2.95–2.93 (dq, 2H, *J* = 11.7 Hz), 2.21 (s, 6H), 2.20–2.17 (dd, 1H, *J* = 12.3, 12.4 Hz), 2.15–2.13 (d, 2H, *J* = 7.0 Hz), 2.00–1.95 (td, 2H, *J* = 11.7, 11.8 Hz), 1.72–1.69 (dq, 2H, *J* = 12.1 Hz), 1.68–1.69 (m, 1H), 1.62–1.59 (m, 2H), 1.23–1.15 (m, 2H). MS (ESI$^+$): m/z 277.15 $[M+1]^+$ 100%.

4-(2,5-Dimethyl-1*H*-pyrrol-1-yl)-1-(4-(trifluoromethyl)phenyl)piperidine (15).

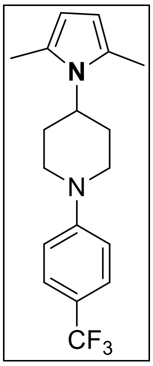

4-(2,5-Dimethyl-1*H*-pyrrol-1-yl)piperidine (0.300 g, 1.68 mmol), 2-Bromo benzotrifluoride (0.378 g, 1.68 mmol), BINAP (0.052 g, 0.084 mmol), tBuONa (0.242 g, 2.52 mmol), and $Pd_2(dppp)Cl_2$ (0.041 g, 0.050 mmol), was mixed,purged with N_2 and anhydrous toluene (10 mL) added, purging with N_2 again. The mixture was heated at 100 °C under N_2 for 5 h, cooled, diluted with ethylacetate (20 mL) andfiltered through a pad of celite^{TM} ,which was washed with ethylacetate (20 mL). The combined organic extracts were washed with aq.$NaHCO_3$. The aqueous layer was back extracted with ethylacetate (2 x 100 mL) andthe combined organic extracts were dried over $MgSO_4$, and concentrated in vacuo to afford (**15**) as a brown oil (0.348 g, 64%). ^{1}H NMR (500 MHz, $CDCl_3$): *δ* ppm. 7.36–7.34 (d, 2H, *J* = 8.6 Hz), 6.93–6.92 (d, 2H, *J* = 8.7 Hz), 5.48 (s, 2H), 4.11–4.05 (tt, 1H, *J* = 12.5 Hz),

3.90–3.84 (dq, 2H, *J* = 13.1 Hz), 2.84–2.79 (td, 2H, *J* = 12.8 Hz), 2.14–2.08 (td, 2H, *J* = 12.6 Hz), 2.06 (s, 6H), 1.71–1.78 (d, 2H, *J* = 13.1 Hz). MS (ESI^+): m/z 323.28 $[M+1]^+$ 100%.

Compounds 12d, 12e, 12s, were prepared following General Procedure B using the appropriate aldehyde, and subsequently used without purification or full characterisation.

Compounds 13b-i, were prepared following General Procedure C using the appropriate aryl/hetero bromide, and subsequently used without purification or full characterisation.

Compounds 14a-d, 14f-i, were prepared following General Procedure D using the appropriate substituted alcohol, and subsequently used without purification or full charcterisation..

E. General procedure for the synthesis of 2,5-dimethyl-1-substituted-3-formylpyrroles: (16a-z, aa-aj)

Phosphorous oxychloride (6 mmol) was added dropwise to ice-cooled N,N-dimethylformamide (12 mL) under an N_2 atmosphere. After stirring at room temperature for 15 min a solution of the pyrrole (**11a, 12a-s, 13a-i, 14a-e, 15**) (1 mmol) in N,N-dimethylformamide (5 mL) was added and the mixture was heated under a N_2 atmosphere (100 ºC, 3 h). After cooling 30% NaOH was added drop-wise to adjust the pH to ~10.0. The resultant solid precipitate was filtered and washed with water, affording the the 2,5-dimethyl-1-substituted 3-formylpyrrole **(16a-z, aa-aj)** (80-95% yield).

2,5-Dimethyl-1-(1-methylpiperidin-4-yl)-1*H*-pyrrole-3-carbaldehyde (16a).

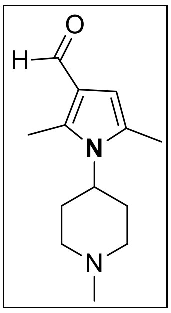

General Procedure E to give (**16a**) as a dark brown oil (0.400 g, 20%). ^{1}H NMR (500 MHz, $CDCl_3$): δ ppm. 9.70 (s, 1H, -C*H*O), 6.18 (s, 1H), 3.94–3.89 (tt, 1H, *J* = 4.0 Hz), 3.01–2.99 (d, 2H, *J* = 11.4 Hz), 2.50 (s, 3H), 2.36–2.33 (dd, 2H, *J* = 12.5 Hz), 2.30 (s, 3H), 2.37 (s, 3H), 2.11–2.06 (t, 2H, *J* = 11.4 Hz), 1.77–1.74 (d, 2H, *J* = 12.4 Hz). MS (ESI$^+$): m/z 221.11 $[M+1]^+$ 100%.

1-(1-Isopentylpiperidin-4-yl)-2,5-dimethyl-1*H*-pyrrole-3-carbaldehyde (16c).

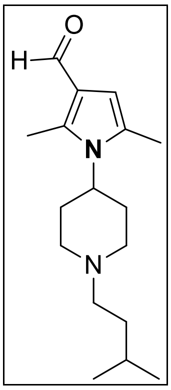

General Procedure E to give (**16c**) as a brown oil (0.051 g, 17%). ^{1}H NMR (500 MHz, $CDCl_3$): δ ppm. 9.72 (s, 1H, -C*H*O), 6.18 (s, 1H), 3.93–3.88 (t, 1H, *J* = 12.2 Hz), 3.06 (br s, 2H), 2.51 (s, 3H), 2.34 (br s, 2H), 2.24 (s, 3H), 2.01 (br s, 2H), 1.77–1.74 (d, 2H, *J* = 12.1 Hz), 1.57–1.49 (m, 3H), 1.36 (br s, 2H), 0.85–0.84 (d, 6H, *J* = 6.6Hz). MS (ESI$^+$): m/z 277.14 $[M+1]^+$ 100%.

2,5-Dimethyl-1-(1-propylpiperidin-4-yl)-1*H*-pyrrole-3-carbaldehyde (16d).

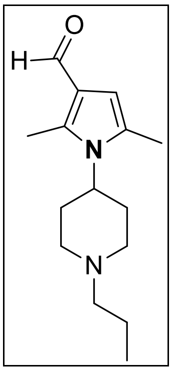

General Procedure E to give (**16d**) as a brown oil (0.192 g, 64%). ^{1}H NMR (500 MHz, $CDCl_3$): δ ppm. 9.73 (s, 1H, -C*H*O), 6.20 (s, 1H), 3.96–3.89 (m, 1H), 3.06–3.04 (d, 2H, *J* = 2.1 Hz), 2.91 (s, 2H), 2.83 (s, 2H), 2.53 (s, 3H), 2.32–2.30 (m, 2H), 2.29 (s, 1H), 2.26 (s, 3H), 2.04–1.99 (td, 1H, *J* = 11.9, 2.2 Hz), 1.78–1.75 (m, 2H), 0.89–0.86 (t, 3H, *J* = 7.3, 7.5 Hz). MS (ESI$^+$): m/z 249.35 $[M+1]^+$ 100%.

2,5-Dimethyl-1-(1-neopentylpiperidin-4-yl)-1*H*-pyrrole-3-carbaldehyde (16e).

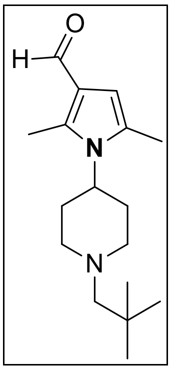

General Procedure E to give (**16e**) as a brown oil (0.125 g, 40%). ^{1}H NMR (500 MHz, $CDCl_3$): *δ* ppm. 9.67 (s, 1H, -C*H*O), 6.13 (s, 1H), 3.86–3.81 (tt, 1H, *J* = 4.2, 4.0 Hz), 2.77 (s, 2H), 2.48 (s, 3H), 2.30–2.25 (td, 2H, *J* = 11.7, 10.7 Hz), 2.21 (s, 3H), 2.20–2.15 (dd, 2H, *J* = 12.0, 12.2 Hz), 2.00 (s, 2H), 1.63–1.60 (m, 2H), 0.79 (s, 9H). MS (ESI$^+$): m/z 277.54 $[M+1]^+$ 100%.

2,5-Dimethyl-1-(1-(pyridin-2-ylmethyl)piperidin-4-yl)-1*H*-pyrrole-3-carbaldehyde (16f).

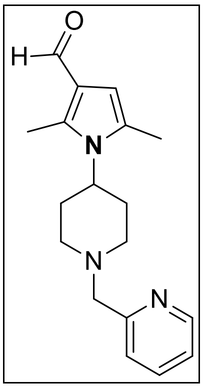

General Procedure E to give (**16f**) as a brown oil (0.083 g, 56%). ^{1}H NMR (500 MHz, $CDCl_3$): *δ* ppm. 9.77 (s, 1H, -C*H*O), 8.58–8.56 (d, 1H, *J* = 1.5 Hz), 7.70–7.66 (tt, 1H, *J* = 7.6 Hz), 7.43–7.41 (d, 1H, *J* = 7.8 Hz), 7.20–7.18 (td, 1H, *J* = 7.8, Hz), 6.24 (s, 1H), 4.02–3.96 (tt, 1H, *J* = 12.5 Hz), 3.55–3.58 (d, 2H, *J* = 3.7 Hz), 3.05–3.03 (dq, 2H, *J* = 9.6 Hz), 2.59 (s, 3H), 2.40–2.34 (m, 2H), 2.32 (s, 3H), 2.18–2.12 (td, 2H, *J* = 11.8 Hz), 1.84–1.81 (dq, 2H, *J* = 12.1 Hz). MS (ESI$^+$): m/z 298.26 $[M+1]^+$ 100%.

2,5-Dimethyl-1-(1-(pyridin-3-ylmethyl)piperidin-4-yl)-1*H*-pyrrole-3-carbaldehyde (16g).

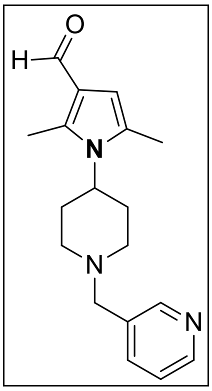

General Procedure E to give (**16g**) as a brown oil (0.08 g, 51%). ^{1}H NMR (500 MHz, $CDCl_3$): *δ* ppm. 9.79 (s, 1H, -C*H*O), 8.58 (d, 1H, *J* = 1.5 Hz), 8.55–8.54 (dd, 1H, *J* = 4.6, 4.8 Hz), 7.71–7.69 (dt, 1H, *J* = 7.7, 7.8 Hz), 7.31–7.30 (td, 1H, *J* = 4.7, 7.8, Hz), 6.27 (s, 1H), 4.02–3.96 (tt, 1H, *J* = 12.5 Hz), 3.55–3.58 (d, 2H, *J* = 3.7 Hz), 3.05–3.03 (dq, 2H, *J* = 9.6 Hz), 2.59 (s, 3H), 2.40–2.34 (m, 2H), 2.32 (s, 3H), 2.18–2.12 (td, 2H, *J* = 11.7, 11.8 Hz), 1.84–1.81 (dq, 2H, *J* = 11.6, 12.1 Hz). MS (ESI$^+$): m/z 298.27 [M+1]$^+$ 100%.

2,5-Dimethyl-1-(1-(2-(trifluoromethyl)benzyl)piperidin-4-yl)-1*H*-pyrrole-3-carbaldehyde (16k).

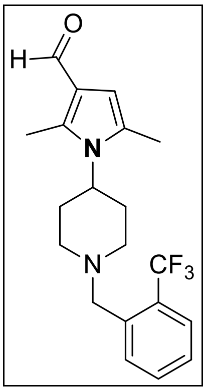

General Procedure E to give (**16k**) as a brown oil (0.2 g, 68%). ^{1}H NMR (500 MHz, $CDCl_3$): *δ* ppm. 9.76 (s, 1H, -C*H*O), 7.80–7.78 (d, 1H, *J* = 7.7 Hz), 7.61–7.60 (d, 1H, *J* = 7.7 Hz), 7.54–7.51 (t, 1H, *J* = 7.5, 7.6 Hz), 7.34–7.31 (t, 1H, *J* = 7.6, 7.5 Hz), 6.23 (s, 1H), 4.04–3.97 (m, 1H), 3.68 (s, 2H), 3.00–2.98 (d, 2H, *J* = 2.0, 7.7 Hz), 2.92 (s, 1H), 2.85 (s, 1H), 2.53 (s, 3H), 2.35–2.32 (m, 1H), 2.30 (s, 3H), 2.21–2.16 (m, 2H), 2.13 (s, 1H). MS (ESI$^+$): m/z 365.14 [M+1]$^+$ 100%.

2,5-Dimethyl-1-(1-(4-(trifluoromethyl)phenyl)piperidin-4-yl)-1*H*-pyrrole-3-carbaldehyde (16l).

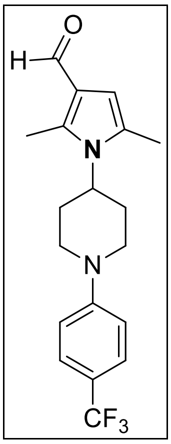

General Procedure E to give (**16l**) as a brown oil (0.250 g, 66%). ^{1}H NMR (500 MHz, $CDCl_3$): δ ppm. 9.79 (s, 1H, -C*H*O), 7.55–7.54 (d, 2H, J = 8.5 Hz), 7.17–7.12 (d, 2H, J = 8.7 Hz), 6.19 (s, 1H), 4.52–4.45 (tt, 1H, J = 12.5 Hz), 4.14–4.10 (dq, 2H, J = 13.1 Hz), 3.14–3.07 (td, 2H, J = 12.8 Hz), 2.59 (s, 3H), 2.43–2.34 (m, 2H), 2.30 (s, 3H), 2.01–1.98 (d, 2H, J = 13.1 Hz). MS (ESI$^+$): m/z 351.14 $[M+1]^+$ 100%.

1-(1-(Cyclopropylmethyl)piperidin-4-yl)-2,5-dimethyl-1*H*-pyrrole-3-carbaldehyde (16u).

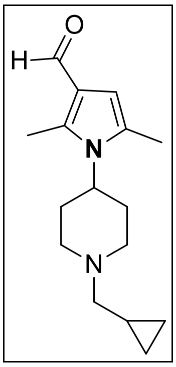

General Procedure E to give (**16u**) as a brown oil (0.130 g, 43%). ^{1}H NMR (500 MHz, $CDCl_3$): δ ppm. 9.66 (s, 1H, -C*H*O), 6.13 (s, 1H), 3.84–3.82 (tt, 1H, J = 3.9 Hz), 3.14–3.12 (q, 2H, J = 2.1, 7.7 Hz), 2.84 (s, 3H), 2.76 (s, 3H), 2.47 (s, 3H), 2.27–2.21 (td, 1H, J = 3.7, 8.8 Hz), 2.02–1.97 (td, 2H, J = 10.0, 11.9 Hz), 1.73–1.70 (d, 2H, J = 1.4 Hz), 0.79–0.73 (m, 1H), 0.44–0.41 (m, 2H), 0.02–0.00 (m, 2H). MS (ESI$^+$): m/z 261.17 $[M+1]^+$ 100%.

1-(1-(Cyclohexylmethyl)piperidin-4-yl)-2,5-dimethyl-1*H*-pyrrole-3-carbaldehyde (16v).

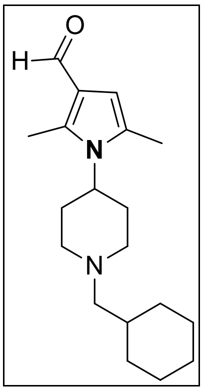

General Procedure E to give (**16v**) as a brown oil (0.146 g, 50%). ^{1}H NMR (500 MHz, $CDCl_3$) : *δ* ppm. 9.64 (s, 1H, -C*H*O), 6.10 (s, 1H), 3.86–3.79 (m, 1H), 2.89–2.87 (d, 2H, *J* = 10.2 Hz), 2.45 (s, 3H), 2.18 (s, 3H), 2.15–2.12 (m, 1H), 2.02–2.01 (d, 2H, *J* = 7.1 Hz), 1.90–1.85 (m, 2H), 1.66–1.64 (d, 4H, *J* = 1.4 Hz), 1.60–1.53 (m, 3H), 1.38–1.30 (m, 2H), 1.15–1.01 (m, 3H), 0.77–0.70 (m, 2H). MS (ESI$^+$): m/z 303.44 $[M+1]^+$ 100%.

2,5-Dimethyl-1-(1-((tetrahydro-2*H*-pyran-4-yl)methyl)piperidin-4-yl)-1*H*-pyrrole-3-carbaldehyde (16w).

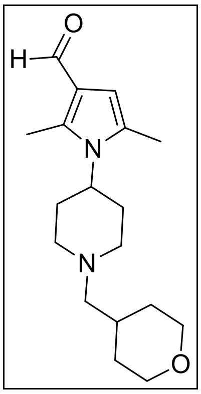

General Procedure E to give (**16w**) as a brown oil (0.05 g, 22%). ^{1}H NMR (500 MHz, $CDCl_3$): *δ* ppm. 9.70 (s, 1H, -C*H*O), 6.17 (s, 1H), 3.91–3.84 (m, 4H), 3.34–3.29 (td, 2H, *J* = 11.9, 11.6 Hz), 2.95–2.93 (dq, 2H, *J* = 11.7 Hz), 2.50 (s, 3H), 2.22 (s, 3H), 2.20–2.17 (dd, 1H, *J* = 12.3, 12.4 Hz), 2.15–2.13 (d, 2H, *J* = 7.0 Hz), 2.00–1.95 (td, 2H, *J* = 11.7, 11.8 Hz), 1.72–1.69 (dq, 2H, *J* = 12.1 Hz), 1.68–1.69 (m, 1H), 1.62–1.59 (m, 2H), 1.23–1.15 (m, 2H). MS (ESI$^+$): m/z 305.15 $[M+1]^+$ 100%.

2,5-Dimethyl-1-(1-((1-methylpiperidin-4-yl)methyl)piperidin-4-yl)-1*H*-pyrrole-3-carbaldehyde (16x).

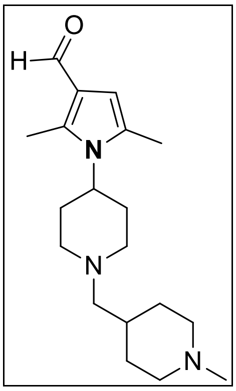

General Procedure E to give (**16x**) as a brown oil (0.095 g, 45%). ^{1}H NMR (500 MHz, $CDCl_3$): *δ* ppm. 9.69 (s, 1H, -C*H*O), 6.18 (s, 1H), 3.92–3.86 (m,1H), 3.62 (br s,1H), 3.36 (s, 2H), 2.96–2.94 (d, 2H, *J* = 9.7 Hz), 2.82–2.80 (m, 2H), 2.51 (s, 3H), 2.24 (s, 3H), 2.21 (s, 3H), 2.15–2.14 (d, 2H, *J* = 7.1 HZ), 1.99–1.93 (m, 2H, *J* = 11.8 Hz), 1.90–1.85 (m, 2H), 1.71–1.69 (br s 4H), 1.45–1.36 (m, 1H), 1.24–1.14 (m, 1H). MS (ESI^{+}): m/z 318.26 $[M+1]^{+}$ 100%.

2,5-Dimethyl-1-(1-((6-(trifluoromethyl)pyridin-3-yl)methyl)piperidin-4-yl)-1*H*-pyrrole-3-carbaldehyde (16y).

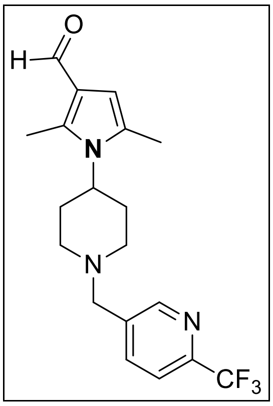

General Procedure E to give (**16y**) as a brown oil (0.18 g, 83%). ^{1}H NMR (500 MHz, $CDCl_3$): *δ* ppm. 9.76 (s, 1H, -C*H*O), 8.66 (d, 1H), 7.88–7.86 (dd, 1H, *J* = 1.4, 6.6 Hz), 7.65–7.64 (d, 1H, *J* = 8.0 Hz), 6.22 (s, 1H), 4.02–3.95 (m, 1H), 3.62 (br s, 2H), 3.00–2.97 (m, 2H), 2.92 (s, 1H), 2.84 (d, 1H, *J* = 0.4 Hz), 2.55 (s, 3H), 2.28 (s, 3H), 2.20–2.15 (m, 2H), 1.81-1.78 (m, 2H). MS (ESI^{+}): m/z 366.15 $[M+1]^{+}$ 100%.

1-(1-(4-Fluoro-2-(trifluoromethyl)benzyl)piperidin-4-yl)-2,5-dimethyl-1*H*-pyrrole-3-carbaldehyde (16z).

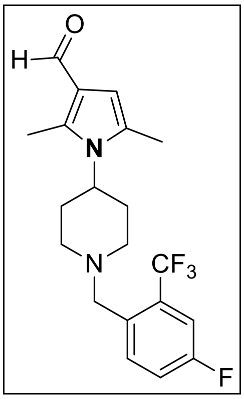

General Procedure E to give (**16z**) as a brown oil (0.150 g, 89%). ^{1}H NMR (500 MHz, $(CD_3)_2CO$): δ ppm. 9.79 (s, 1H, -C*H*O), 7.98 (br s, 2H), 7.50–7.46 (m, 1H), 6.18 (s, 1H), 4.24–4.21 (m, 1H), 3.71 (s, 2H), 3.02–3.00 (d, 2H, *J* = 9.3 HZ), 2.68 (s, 3H), 2.61 (s, 3H), 2.39–2.36 (m, 1H), 2.34 (s, 1H), 2.32–2.27 (m, 2H), 1.86–1.84 (d, 2H, *J* = 3.6 Hz). MS (ESI^{+}): m/z 383.15 $[M+1]^{+}$ 100%.

2,5-Dimethyl-1-(1-((1-methyl-1*H*-pyrazol-3-yl)methyl)piperidin-4-yl)-1*H*-pyrrole-3-carbaldehyde (16aa).

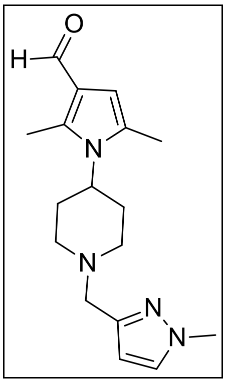

General Procedure E to give (**16aa**) as a brown oil (0.100 g, 60% yield). ^{1}H NMR (500 MHz, $CDCl_3$): δ ppm. 9.72 (s, 1H, -C*H*O), 7.24–7.23 (d, 1H, *J* = 2.1 Hz), 7.19 (s, 1H), 6.17 (d, 1H, *J* = 2.1 Hz), 3.90–3.81 (m, 1H), 3.81 (br s, 3H), 3.53 (br s 2H), 3.05–3.03 (d, 2H, *J* = 11.0 Hz), 2.49 (s, 3H), 2.28–2.25 (m, 2H), 2.22 (s, 3H), 2.09–2.04 (m, 2H), 1.73–1.70 (s, 2H, *J* = 6.8 Hz). MS (ESI^{+}): m/z 301.21 $[M+1]^{+}$ 100%.

1-(1-((1*H*-Imidazol-2-yl)methyl)piperidin-4-yl)-2,5-dimethyl-1*H*-pyrrole-3-carbaldehyde (16ab).

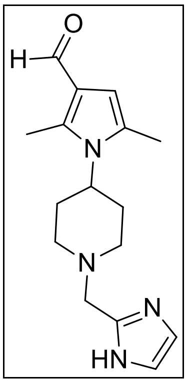

General Procedure E to give (**16ab**) as a brown oil (0.100 g, 36%). ^{1}H NMR (500 MHz, $CDCl_3$): *δ* ppm. 9.80 (s, 1H, -C*H*O), 7.05 (s, 2H), 6.28 (s, 1H), 4.04–3.95 (m, 1H), 3.73 (s, 2H), 3.05–3.02 (d, 2H, *J* = 10.3 Hz), 2.60 (s, 3H), 2.35 (s, 1H), 2.32 (s, 3H), 2.29–2.24 (m, 2H), 2.19 (s, 1H), 1.86–1.83 (m, 2H). MS (ESI$^+$): m/z 287.20 $[M+1]^+$ 100%.

2,5-Dimethyl-1-(1-((5-methylisoxazol-3-yl)methyl)piperidin-4-yl)-1*H*-pyrrole-3-carbaldehyde (16ac).

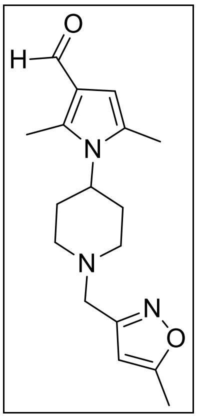

General Procedure E to give (**16ac**) as a brown oil (0.089 g, 38%). ^{1}H NMR (500 MHz, $CDCl_3$): *δ* ppm. 9.80 (s, 1H, -C*H*O), 6.27 (d, 1H, *J* = 0.8 Hz), 6.04–6.03 (d, 1H, *J* = 0.7 Hz), 4.00–3.95 (m, 1H), 3.63 (s, 2H), 3.07–3.05 (m, 2H), 2.98 (s, 1H), 2.90 (d, 1H, *J* = 0.5 Hz), 2.59 (s, 3H), 2.45 (s, 3H), 2.37–2.34 (m, 1H), 2.32 (s, 3H), 2.24–2.22 (dd, 1H, *J* = 9.8 Hz), 1.84–1.81 (m, 2H), 1.73 (s, 1H). MS (ESI$^+$): m/z 302.20 $[M+1]^+$ 100%.

2,5-Dimethyl-1-(1-(oxazol-4-ylmethyl)piperidin-4-yl)-1*H*-pyrrole-3-carbaldehyde (16ad).

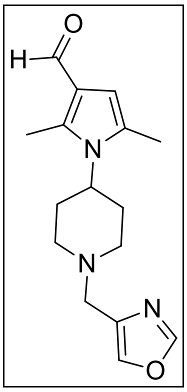

General Procedure E to give (**16ad**) as a brown oil (0.068 g, 27%). ^{1}H NMR (500 MHz, $CDCl_3$): δ ppm. 9.77 (s, 1H, -C*H*O), 7.99 (s, 1H), 7.87 (s, 1H), 6.22 (s, 1H), 3.97–3.91 (m, 1H), 3.56 (s, 2H), 3.12–3.07 (d, 2H, *J* = 1.9, 7.9 Hz), 2.94 (s, 3H), 2.86 (s, 3H), 2.55 (s, 2H), 2.20–2.15 (m, 2H), 1.81–1.78 (d, 2H, *J* = 1.7, 10.4 Hz). MS (ESI^+): m/z 288.10 $[M+1]^+$ 100%.

2,5-Dimethyl-1-(1-((1-methyl-1*H*-imidazol-2-yl)methyl)piperidin-4-yl)-1*H*-pyrrole-3-carbaldehyde (16ae).

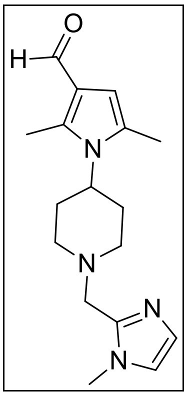

General Procedure E to give (**16ae**) as a brown oil (0.200 g, 64%). ^{1}H NMR (500 MHz, $CDCl_3$): δ ppm. 9.70 (s, 1H, -C*H*O), 6.86 (s, 1H), 6.80 (s, 1H), 6.17 (s, 1H), 3.66 (s, 3H), 3.57 (br s, 2H), 2.93–2.91 (dd, 2H, *J* = 9.5 Hz), 2.49 (s, 3H), 2.21(s, 3H), 2.18–2.17 (d, 1H, *J* = 3.00 Hz), 2.15 (s, 1H), 2.13 (d, 1H, *J* = 1.53 Hz), 1.75–1.72(dd, 1H, *J* = 3.9 Hz), 1.18 (br s, 3H). MS (ESI^+): m/z 301.00 $[M+1]^+$ 100%.

2,5-Dimethyl-1-(1-((1-methyl-1*H*-pyrrol-2-yl)methyl)piperidin-4-yl)-1*H*-pyrrole-3-carbaldehyde (16af).

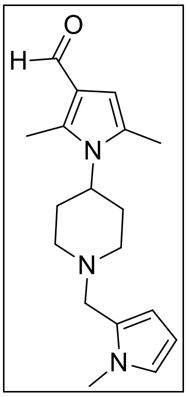

General Procedure E to give (**16af**) as a brown oil (0.116 g, 48%). ^{1}H NMR (500 MHz, $CDCl_3$): *δ* ppm. 9.76 (s, 1H, -C*H*O), 7.99 (s, 1H), 6.60–6.59 (m, 1H), 6.23 (s, 1H), 6.03–6.01 (m, 1H), 5.94–5.97 (m, 2H), 3.45 (s, 1H), 3.05–3.01 (d, 2H, *J* = 3.5 Hz), 2.94 (s, 3H), 2.86 (s, 3H), 2.55 (br s, 2H), 2.28 (s, 3H), 2.04 (d, 2H, *J* = 3.7 Hz), 1.80–1.76 (d, 2H, *J* = 3.0, 1.9 Hz). MS (ESI$^+$): m/z 300.01 [M+1]$^+$ 100%.

2,5-Dimethyl-1-(1-(4-(5-methyl-1,2,4-oxadiazol-3-yl)benzyl)piperidin-4-yl)-1*H*-pyrrole-3-carbaldehyde (16ag).

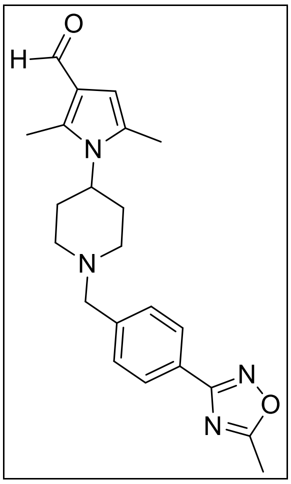

General Procedure E to give (**16ag**) as a brown oil (0.085 g, 52%). ^{1}H NMR (500 MHz, $CDCl_3$): *δ* ppm. 9.81 (s, 1H, -C*H*O), 8.05 (d, 2H, *J* = 1.7, 4.8 Hz), 7.48–7.46 (d, 2H, *J* = 8.3 Hz), 6.28 (s, 1H), 4.03–3.96 (m, 1H), 3.63 (s, 2H), 3.08–3.06 (d, 2H, *J* = 2.0, 7.6 Hz), 2.97 (s, 3H), 2.90 (s, 3H), 2.68 (s, 3H), 2.61 (s, 2H), 2.18–2.13 (m, 2H), 1.84–1.81 (m, 2H). MS (ESI$^+$): m/z 379.20 [M+1]$^+$ 100%.

2,5-Dimethyl-1-(1-((6-morpholinopyridin-3-yl)methyl)piperidin-4-yl)-1*H*-pyrrole-3-carbaldehyde (16ah).

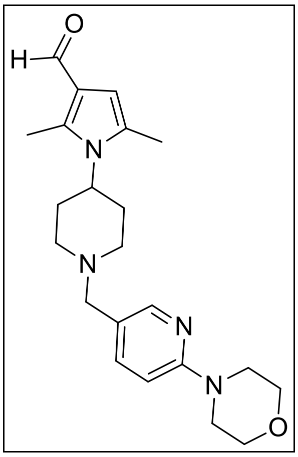

General Procedure E to give (**16ah**) as a brown oil (0.118 g, 52%). ^{1}H NMR (500 MHz, $CDCl_3$): *δ* ppm. 9.79 (s, 1H, -C*H*O), 8.13–8.12 (d, 1H, *J* = 2.0 Hz), 7.53–7.51 (dd, 1H, *J* = 2.1, 6.5 Hz), 6.68–6.65 (dd, 1H, *J* = 3.0, 5.6 Hz), 6.27 (s, 1H), 4.00–3.93 (m, 1H), 3.86–3.83 (m, 4H), 3.53–3.51 (m, 4H), 3.47 (br s, 2H),. 3.05–3.03 (d, 2H, *J* = 11.6 Hz), 2.59 (s, 3H), 2.31 (s, 3H), 2.30–2.26 (m, 1H), 2.19 (s, 1H), 2.11–2.07 (m, 2H), 1.82–1.79 (m, 2H). MS (ESI^+): m/z 383.15 $[M+1]^+$ 100%.

2,5-Dimethyl-1-(1-((2-morpholinopyrimidin-5-yl)methyl)piperidin-4-yl)-1*H*-pyrrole-3-carbaldehyde (16ai).

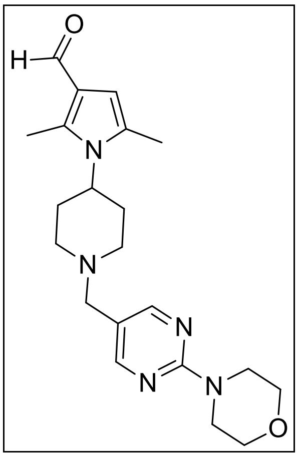

General Procedure E to give (**16ai**) as a brown oil (0.092 g, 26%). ^{1}H NMR (500 MHz; $CDCl_3$) *δ* ppm. 9.71 (s, 1H, -C*H*O), 8.20 (s, 2H), 6.18 (s, 1H), 3.92–3.85 (m, 1H), 3.73–3.70 (m, 8H), 3.33 (s, 2H), 2.97–2.94 (d, 2H, *J* = 11.4 Hz), 2.50 (s, 3H), 2.22 (s, 3H), 2.18 (s, 1H), 2.04–1.99 (d, 2H, *J* = 11.4 Hz), 1.97 (s, 1H), 1.74–1.71 (d, 2H, *J* = 1.7, 10.4 Hz). MS (ESI^+): m/z 384.20 $[M+1]^+$ 100%.

Compounds 16b, 16h-j, 16m-t, 16aj were prepared following General Procedure E and subsequently used without full charcterisation.

F. General procedure for condensation of 3 with 3-formyl pyrroles:

To a solution of ethyl 5-methyl-3-oxo-1,2-dihydropyrrole-4-carboxylate **(3)** (1.0 equiv) in absolute ethanol (3 mL) was added 2,5-dimethyl-1-aryl/substituted aryl-3-formylpyrroles **(7)** (1.0 equiv) and potassium hydrogen sulphate (0.2 equiv). The mixture was heated at 70–80 ºC for 3 h, poured into crushed ice and filtered to afford the desired product.

(*E*)-Ethyl 5-((2,5-dimethyl-1-(2-(trifluoromethyl)phenyl)-1*H*-pyrrol-3-yl)methylene)-2-methyl-4-oxo-4,5-dihydro-1*H*-pyrrole-3-carboxylate (**TDR32750**).

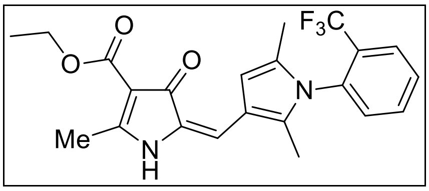

Yellow powder (0.22 g, 89%), mp 230–235 ºC. ^{1}H NMR (500 MHz, DMSO-d_6): δ 10.3 (s, 1H, -NH), 8.01 (t, 1H, J = 7.7 Hz) 7.91 (t, 1H) , 7.8 (t, 1H, J = 7.7 Hz), 7.51 (t, 1H, J = 7.7 Hz), 6.71 (s, 1H), 6.69 (s, 1H), 4.13-4.09 (q, 2H, J = 6.95), 2.58 (s, 3H), 1.99 (s, 3H), 1.90 (s, 3H), 1.23 (t, 3H, J = 7.10 Hz). ^{13}C NMR (125 MHz, DMSO-d_6): δ 180.58, 170.23, 163.33, 135.97, 134.36 (2C), 131.67, 131.60, 130.43 (2C), 129.17 (2C), 127.51, 113.55, 169.76, 105.95, 102.44, 58.25, 15.79, 14.40, 11.96, 10.30. IR (KBr) v 3500-2000 (max at 3176.47, 2926.66, and 2340.11 N-H, and C-H st), 1661.80 and 1583.65 (C=O, ar-C-C and ar-C-N st) cm^{-1}. HRMS (*m/z*): [MH$^+$] calcd for $C_{22}H_{22}N_2O_3F_3$, 419.1577; found 419.1566.

(*E*)-Ethyl 5-((1-(1-benzylpiperidin-4-yl)-2,5-dimethyl-1*H*-pyrrol-3-yl)methylene)-2-methyl-4-oxo-4,5-dihydro-1*H*-pyrrole-3-carboxylate (17).

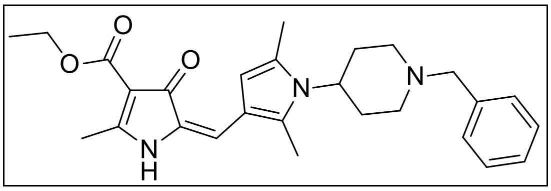

Yellow powder (0.100 g, 40%), mp 150–155 ºC. ^{1}H NMR (500 MHz, CD_3OD): δ 10.13 (s, 1H, -N*H*), 7.36–7.30 (m, 5H), 6.65 (s, 1H), 6.51 (s, 1H), 4.12–4.07 (m, 3H), 3.55 (br s, 2H),

2.96–2.94 (d, 2H, J = 7.0 Hz), 2.53 (s, 3H), 2.44–2.42 (t, 2H, J = 5.7 Hz), 2.36 (s, 3H), 2.32 (s, 3H), 2.18–2.14 (d, 2H, J = 9.1 Hz), 1.76–1.74 (d, 2H, J = 10.4, Hz), 1.22–1.19 (t, 3H, J = 7.1 Hz). ^{13}C-NMR (125 MHz, DMSO-d$_6$): 180.3, 178.3, 169.2, 164.2, 163.7, 163.3, 134.6, 130.1, 128.8, 128.5, 128.1, 126.9, 117.4, 113.0, 110.1, 102.4, 64.0, 61.7, 58.1, 57.8, 55.1, 54.6, 52.6, 30.5, 15.7, 14.4, 11.0. HRMS (*m/z*): [MH+] calcd for $C_{27}H_{34}N_3O_3$, 448.2469; found 448.2468.

(*E*)-Ethyl 5-((2,5-dimethyl-1-(piperidin-4-yl)-1*H*-pyrrol-3-yl)methylene)-2-methyl-4-oxo-4,5-dihydro-1*H*-pyrrole-3-carboxylate (18).

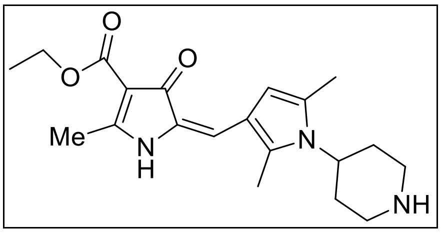

YYellow powder (0.100 g, 40%), mp >250 ºC. ^{1}H NMR (500 MHz, CD_3OD): δ 10.25 (s, 1H, -N*H*), 9.71 (s, 1H, -N*H*), 6.65 (s, 1H), 6.59 (s, 1H), 4.10–4.09 (m, 2H), 3.76–3.74 (m, 1H), 3.11–3.06 (m, 2H, J = 12.5 Hz), 2.56 (s, 2H), 2.51 (s, 3H), 2.40 (s, 3H), 2.34 (s, 3H), 1.91–1.89 (d, 2H, J = 8.9 Hz), 1.41–1.39 (d, 2H, J = 6.5, Hz), 1.22–1.20 (t, 3H, J = 7.1 Hz). ^{13}C-NMR (125 MHz, DMSO-d$_6$): 180.3, 178.3, 169.2, 128.1, 126.9, 117.4, 113.0, 110.1, 102.4, 64.0, 61.7, 58.1, 57.8, 55.1, 54.6, 52.6, 30.5, 15.7, 14.4, 11.0. HRMS (*m*/*z*): [MH$^+$] calcd for $C_{20}H_{28}N_3O_3$, 358.6964; found 358.6824.

(E)-Ethyl 5-((2,5-dimethyl-1-(1-methylpiperidin-4-yl)-1*H*-pyrrol-3-yl)methylene)-2-methyl-4-oxo-4,5-dihydro-1*H*-pyrrole-3-carboxylate (**19**).

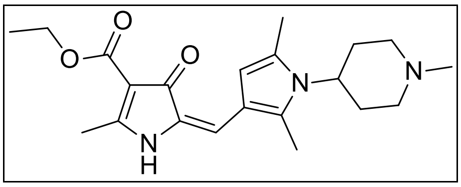

Yellow powder (0.016 g, 19%), mp 130–135 ºC. ^{1}H NMR (500 MHz, DMSO-d$_6$): δ 10.14 (s, 1H, -N*H*), 6.65 (s, 1H), 6.49 (s, 1H), 4.12–4.06 (m, 2H), 2.98 (br s,1H), 2.57 (s, 3H), 2.51 (s, 3H), 2.44–2.42 (d, 2H, J = 5.8 Hz), 2.35 (s, 3H), 2.29 (s, 3H), 2.26 (br s, 2H), 2.22 (br s, 2H), 1.76–1.75 (d, 2H, J = 5.0 Hz), 1.23–1.21 (t, 3H, J = 1.5, 5.6 Hz). ^{13}C-NMR (125 MHz; DMSO-d$_6$): 180.3, 178.3, 169.3, 163.7, 134.6, 130.1, 128.5, 117.4, 113.1, 110.0, 102.4, 64.0, 58.1, 54.7, 45.2, 30.0, 15.9, 15.7, 15.0, 14.43 11.0. HRMS (*m/z*): [MH+] calcd for $C_{21}H_{30}N_3O_3$, 372.2282; found 372.2297.

(*E*)-Ethyl 5-((1-(1-ethylpiperidin-4-yl)-2,5-dimethyl-1*H*-pyrrol-3-yl)methylene)-2-methyl-4-oxo-4,5-dihydro-1*H*-pyrrole-3-carboxylate (20).

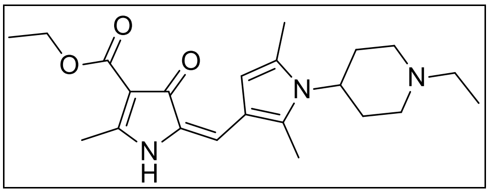

Yellow powder (0.010 g, 40%), mp 140–145 ºC. ^{1}H NMR (500 MHz, DMSO-d$_6$): δ 10.25 (s, 1H, -N*H*), 6.65 (s, 1H), 6.59 (s, 1H), 4.10–4.09 (m, 2H), 3.51 (s, 1H), 3.83–3.81 (d, 2H, *J* = 11.2 Hz), 3.76–3.74 (m, 2H), 3.01 (br s, 2H), 2.56 (s, 3H), 2.51 (s, 3H), 2.40 (br s, 2H), 2.34 (s, 3H), 1.41–1.34 (d, 2H), 1.21 (t, 3H, *J* = 7.1 Hz). 1.01 (t, 3H, *J* = 7.1 Hz). HRMS (*m/z*): [MH+] calcd for $C_{22}H_{31}N_3O_3$, 386.2438; found 386.2439.

(*E*)-ethyl 5-((1-(1-isopentylpiperidin-4-yl)-2,5-dimethyl-1*H*-pyrrol-3-yl)methylene)-2-methyl-4-oxo-4,5-dihydro-1*H*-pyrrole-3-carboxylate (21).

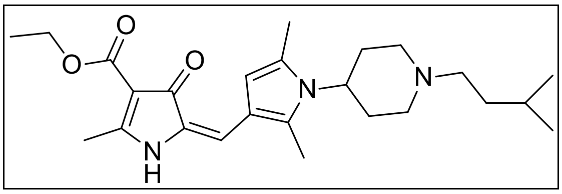

Yellow powder (0.026 g, 89%), mp 158–162 ºC. ^{1}H NMR (500 MHz, DMSO-d$_6$): δ 10.15 (s, 1H, -N*H*), 6.64 (s, 1H), 6.54 (s, 1H), 4.10–4.09 (m, 2H), 3.74–3.73 (m, 1H), 3.46–3.43 (m, 2H), 2.57 (s, 3H), 2.43 (s, 2H), 2.39 (s, 3H), 2.33 (s, 3H), 1.88–1.84 (dd, 2H, *J* = 9.3 Hz), 1.62–1.59 (m, 2H), 1.48 (br s, 2H), 1.36 (s, 2H), 1.26 (s, 1H), 1.23–1.20 (m, 3H), 0.91–0.90 (d, 6H, *J* = 6.5 Hz).^{13}C NMR (125 MHz, DMSO-d$_6$): 180.5, 170.2, 163.3, 135.9, 134.36 (2C), 131.6, 131.6, 130.43 (2C), 129.17 (2C), 127.5, 113.5, 169.7, 105.9, 102.4, 58.2, 44.4, 15.7, 14.4(2C), 11.9(2C), 10.3. HRMS (*m/z*): [MH+] calcd for $C_{25}H_{38}N_3O_3$, 428.2908; found 428.2905.

(*E*)-Ethyl 5-((2,5-dimethyl-1-(1-propylpiperidin-4-yl)-1*H*-pyrrol-3-yl)methylene)-2-methyl-4-oxo-4,5-dihydro-1*H*-pyrrole-3-carboxylate (22).

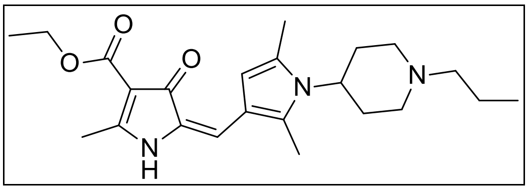

Yellow powder (0.010 g, 14%), mp 145–148 ºC. ^{1}H NMR (500 MHz, CD_3OD): δ 7.13 (s, 1H), 6.51 (s, 1H) , 4.27–4.24 (m, 4H), 4.07–4.04 (m, 1H), 2.62–2.59 (m, 1H), 2.48 (s, 3H), 2.46 (s, 2H), 2.44 (s, 2H), 2.42–2.41 (s, 1H, *J* =1.5 Hz), 2.39 (s, 3H), 2.33 (s, 3H), 1.93–1.91 (d, 2H, *J* = 9.7 Hz), 1.44 (br s, 2H), 1.36–1.33 (t, 3H, *J* = 7.1 Hz), 1.00–0.95 (t, 3H, *J* = 6.5 Hz). HRMS (*m/z*): [MH+] calcd for $C_{23}H_{34}N_3O_3$, 400.2595; found 400.2595.

(*E*)-Ethyl 5-((2,5-dimethyl-1-(1-neopentylpiperidin-4-yl)-1*H*-pyrrol-3-yl)methylene)-2-methyl-4-oxo-4,5-dihydro-1*H*-pyrrole-3-carboxylate (23).

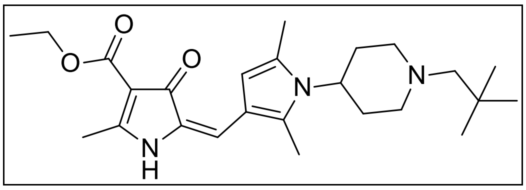

Yellow powder (0.060 g, 77%), mp 139–142 ºC. ^{1}H NMR (500 MHz, CD_3OD): δ 7.00 (s, 1H) , 6.40 (s, 1H) , 5.39 (s,1H), 4.14–4.13 (m, 4H), 3.51–3.44 (m, 2H), 2.89 (s, 3H), 2.76–2.72 (m, 2H), 2.52 (s, 3H), 2.38 (s, 3H), 2.46 (s, 3H), 1.82–1.79 (d, 2H, *J* = 7.0 Hz), 1.24–1.21 (t, 3H, *J* = 7.1 Hz), 0.96 (t, 9H). HRMS (*m/z*): [MH+] calcd for $C_{25}H_{38}N_3O_3$, 428.2908; found 428.2906.

(*E*)-Ethyl 5-((2,5-dimethyl-1-(1-(pyridin-3-ylmethyl)piperidin-4-yl)-1*H*-pyrrol-3-yl)methylene)-2-methyl-4-oxo-4,5-dihydro-1*H*-pyrrole-3-carboxylate (24).

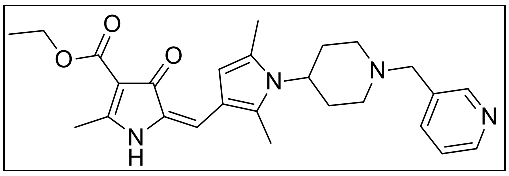

Yellow powder (0.10 g, 83%), mp 129–132 ºC. ^{1}H NMR (500 MHz, DMSO-d_6): δ 10.15 (s, 1H, -N*H*), 8.53–8.52 (d, 1H, *J* = 1.3 Hz), 8.49–8.48 (dd, 1H, *J* = 1.3, 4.8 Hz), 7.75–7.74 (d, 1H, *J* = 7.8 Hz), 7.39–7.36 (dd, 1H, *J* = 4.8, 2.8 Hz), 6.64 (s, 1H), 6.51 (s, 1H), 4.12–4.07 (m, 2H), 4.03–4.01 (m, 2H), 3.57 (br s, 2H), 2.93–2.92 (d, 2H, *J* = 1.9 Hz), 2.95 (s, 3H), 2.37 (s, 1H), 2.31(s, 3H), 2.18–2.12 (m, 3H), 2.26–2.40 (m, 2H), 1.75–1.72 (m, 2H), 1.22–

1.19 (m, 3H). ^{13}C NMR (125 MHz, DMSO-d$_6$): 180.3, 171.4, 169.2, 163.3, 150.0, 148.2, 136.5, 133.5, 130.1, 129.8, 128.5, 123.4, 113.0, 110.1, 107.2, 102.5, 102.4, 58.8, 58.1, 54.5, 52.6, 50.7, 30.5, 14.4, 14.1, 11.0. HRMS (*m/z*): [MH+] calcd for $C_{26}H_{33}N_4O_3$, 449.2534; found 449.2541.

(*E*)-Ethyl 5-((2,5-dimethyl-1-(1-(pyridin-2-ylmethyl)piperidin-4-yl)-1*H*-pyrrol-3-yl)methylene)-2-methyl-4-oxo-4,5-dihydro-1*H*-pyrrole-3-carboxylate (**25**).

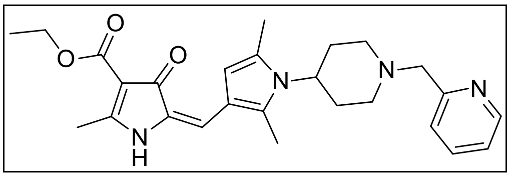

Yellow powder (0.019 g, 15%), mp 118–121ºC. ^{1}H NMR (500 MHz, DMSO-d$_6$): δ 10.14 (s, 1H, -N*H*), 8.51–8.50 (m, 1H), 7.81–7.77 (m, 1H), 7.49–7.48 (d, 1H, *J* = 7.8 Hz), 7.28–7.26 (m, 1H, *J* = 4.9, 1.0 Hz), 6.64 (s, 1H), 6.51 (s, 1H), 4.12–4.02 (m, 2H), 4.06–4.01 (m, 2H), 3.65 (br s, 2H), 2.97–2.95 (d, 2H, *J* = 7.3 Hz), 2.55 (s, 3H), 2.37 (s, 1H), 2.39 (s, 3H), 2.33 (s, 3H), 2.30–2.26 (m, 2H), 2.21–2.20 (m, 2H), 1.76–1.75 (m, 2H), 1.23–1.20 (m, 3H). ^{13}C NMR (125 MHz, DMSO-d$_6$): 180.3, 178.3, 169.2, 164.2, 163.6, 158.4, 148.7, 136.5, 130.1, 129.8, 128.5, 122.7, 122.6, 122.1, 117.4, 110.0, 102.5, 102.4, 58.0, 57.8, 54.6, 53.0, 30.6, 15.7, 14.5, 14.4. HRMS (*m/z*): [MH+] calcd for $C_{26}H_{33}N_4O_3$, 449.2547; found: 449.2539.

(*E*)-Ethyl 5-((2,5-dimethyl-1-(1-(2-(trifluoromethyl)benzyl)piperidin-4-yl)-1*H*-pyrrol-3-yl)methylene)-2-methyl-4-oxo-4,5-dihydro-1*H*-pyrrole-3-carboxylate (**29**).

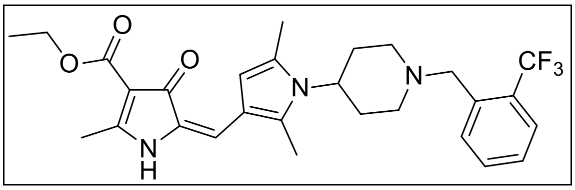

Yellow powder (0.03 g, 45%), mp 135–140 ºC. ^{1}H NMR (500 MHz, CD_3OD): δ 10.3 (s, 1H, -N*H*), 7.86–7.85 (d, 1H, *J* = 7.7 Hz) 7.70–7.68 (d, 1H, *J* = 7.7 Hz), 7.63–7.61 (t, 1H, *J* = 7.7 Hz), 7.46–7.44 (t, 1H, *J* = 7.7 Hz), 6.48 (s, 1H), 4.24–4.23 (m, 2H), 4.18–4.13 (m, 1H), 3.77 (br s, 2H), 3.33–3.32 (q, 2H, *J* = 1.6 Hz), 3.06–3.01 (m, 2H), 2.87 (s, 1H), 2.46 (br s, 3H), 2.37 (br s, 3H), 2.35 (s, 3H), 2.32 –2.28 (m, 2H), 1.82–1.80 (d, 2H, *J* = 12.9, Hz), 1.34–1.31 (t, 3H, *J* = 7.0 Hz). ^{13}C NMR (125 MHz, DMSO-d$_6$): 180.15, 169.8, 165.8, 138.6, 133.33(2C), 132.8, 131.92(2C), 128.53(2C), 127.1, 126.8, 124.9, 116.2, 104.5, 60.35(2C),

59.22(2C), 56.5, 54.70(2C), 36.9, 31.9, 31.6, 16.2, 14.8. HRMS (*m/z*): [MH+] calcd for $C_{28}H_{33}F_3N_3O_3$, 516.2469; found 516.2468.

(*E*)-Ethyl 5-((2,5-dimethyl-1-(1-(4-(trifluoromethyl)phenyl)piperidin-4-yl)-1*H*-pyrrol-3-yl)methylene)-2-methyl-4-oxo-4,5-dihydro-1*H*-pyrrole-3-carboxylate (30).

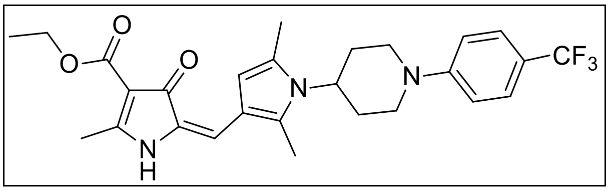

Yellow powder (0.03 g, 42%), mp 153–158 ºC. ^{1}H NMR (500 MHz, Acetone-d$_6$): δ 10.12(s, 1H, -N*H*), 7.83–7.82 (d, 1H, *J* = 9.4 Hz), 7.40–7.38 (t, 1H, *J* = 9.0 Hz), 7.03–7.01 (t, 1H, *J* = 7.7 Hz), 6.66–6.65 (d, 1H, *J* = 7.5 Hz), 6.64 (s, 1H), 6.52 (s,1H), 4.40–4.32 (m, 1H), 4.12–4.08 (m, 2H), 4.06–4.03 (d, 2H, *J* = 13.4 Hz), 3.05–3.00 (t, 2H, *J* = 12.4, 11.3 Hz), 2.56 (s, 3H), 2.36 (s, 3H), 2.28 (s, 3H), 2.20–2.14 (td, 2H, *J* = 12.4, 12.4 Hz), 1.87–1.85 (d, 2H, *J* = 10.4 Hz), 1.23–1.20 (t, 3H, *J* = 7.1 Hz). ^{13}C NMR (125 MHz, DMSO-d$_6$): 180.3, 169.3, 163.3, 162.2, 152.5, 130.0, 128.5, 126.3, 126.2, 126.0, 123.9, 117.5, 117.2, 114.3, 110.0, 102.4, 58.0, 54.2, 47.3, 35.7, 30.7, 29.7, 28.3, 15.7, 14.4, 13.8, 11.0. HRMS (*m/z*): [MH+] calcd for $C_{27}H_{31}F_3N_3O_3$, 502.2312; found 502.2293.

(*E*)-Ethyl 5-((2,5-dimethyl-1-(1-(1-(4-(trifluoromethyl)phenyl)ethyl)piperidin-4-yl)-1*H*-pyrrol-3-yl)methylene)-2-methyl-4-oxo-4,5-dihydro-1*H*-pyrrole-3-carboxylate (35).

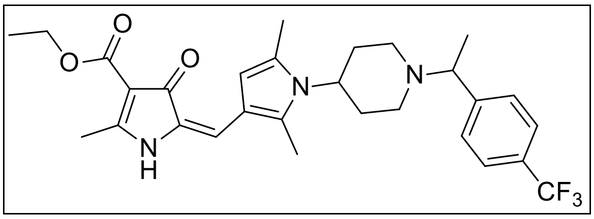

Yellow powder (0.22 g, 89%), mp 130–135 ºC. ^{1}H NMR (500 MHz, CD$_3$OD): δ 7.68–7.66 (d, 2H, *J* = 8.1 Hz), 7.59–7.58 (d, 2H, *J* = 8.0 Hz), 7.1 (t, 1H) , 6.47 (s, 1H), 4.26–4.22 (m, 2H, *J* = 7.1 Hz), 4.12–4.06 (m, 1H), 3.74–3.72 (d, 2H, *J* = 6.6 Hz), 3.03–3.00 (d, 2H, *J* = 12.0 Hz), 2.65 (s, 3H), 2.52–2.51 (d, 1H, *J* = 4.1 Hz), 2.44 (s, 3H), 2.35 (s, 3H), 2.29–2.23 (m, 2H), 1.87–1.84 (d, 1H, *J* = 12.7 Hz), 1.78–1.76 (d, 1H, *J* =11.5 Hz), 1.79–1.48 (d, 3H, *J* = 6.7 Hz), 1.34–1.32 (t, 3H, J = 7.1 Hz). ^{13}C NMR (125 MHz, CD$_3$OD): 183.9, 169.8, 165.8, 148.1, 138.5, 132.8, 129.9, 129.6, 129.6, 126.8, 126.3, 124.6, 116.2, 115.4, 108.4, 65.8,

65.4, 31.5, 60.3, 56.5, 51.6, 51.5, 31.9, 31.8, 19.3, 16.2, 15.5, 14.8, 11.4. HRMS (*m/z*): [MH+] calcd for $C_{29}H_{35}F_3N_3O_3$, 530.2625; found 530.2600.

(*E*)-Ethyl 5-((2,5-dimethyl-1-(1-(1-(2-(trifluoromethyl)phenyl)ethyl)piperidin-4-yl)-1*H*-pyrrol-3-yl)methylene)-2-methyl-4-oxo-4,5-dihydro-1*H*-pyrrole-3-carboxylate (36).

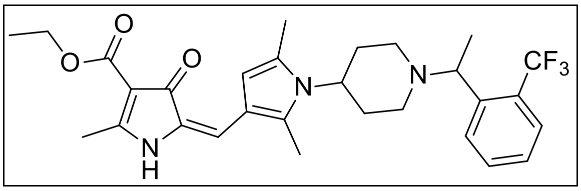

Yellow powder (0.015 g, 23%), mp 138–142 ºC. ^{1}H NMR (500 MHz, CD_3OD): δ 7.60–7.57 (d, 1H, *J* = 9.4 Hz), 7.55–7.54 (t, 1H, *J* = 9.0 Hz), 7.49–7.46 (t, 1H, *J* = 7.75, 6.3 Hz), 7.45–7.42 (d, 1H, *J* = 7.5 Hz), 6.99 (t, 1H) , 6.39 (s, 1H), 4.15–4.10 (m, 2H), 3.98–3.96 (m, 1H), 3.61–3.58 (d, 1H, *J* = 6.7 Hz), 2.90–2.88 (d, 1H, *J* = 10.4 Hz), 2.51 (s, 3H), 2.40–2.39 (d, 1H, *J* = 7.8 Hz), 2.33 (s, 3H), 2.25 (s, 3H), 2.14–2.10 (m, 2H), 2.04–2.00 (m, 1H), 1.76–1.74 (d, 1H, *J* = 11.3 Hz), 1.67–1.65 (d, 1H, *J* =12.2 Hz), 1.37–1.36 (d, 3H, *J* = 6.8 Hz), 1.23–1.20 (t, 3H, *J* = 7.1 Hz). ^{13}C NMR (125 MHz, CD_3OD): 183.9, 169.8, 165.8, 145.2, 138.5, 132.8, 131.8, 130.3, 129.9, 126.8, 125.5, 125.2, 124.6, 116.2, 115.3, 108.4, 104.5, 65.9, 65.4, 60.3, 57.1, 51.8, 31.8, 30.1, 19.3, 16.2, 15.4, 14.8, 11.4. HRMS (*m/z*): [MH+] calcd for $C_{29}H_{35}F_3N_3O_3$, 530.2625; found: 530.2623.

(*E*)-Ethyl 5-((2,5-dimethyl-1-(1-(2-morpholinoethyl)piperidin-4-yl)-1*H*-pyrrol-3-yl)methylene)-2-methyl-4-oxo-4,5-dihydro-1*H*-pyrrole-3-carboxylate (37)

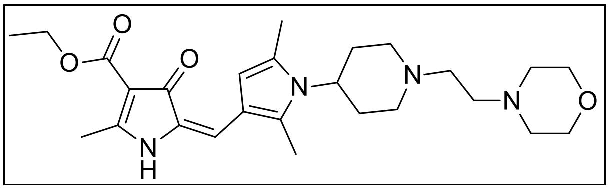

Yellow powder (0.191 g, 65%), mp 115–118 ºC. ^{1}H NMR (500 MHz, CD_3OD): δ 7.13 (s, 1H) , 6.50 (s, 1H), 4.23–4.16 (m, 2H), 4.12–4.06 (m, 1H), 3.73–3.71 (m, 4H), 3.23–3.22 (d, 2H, *J* = 8.1 Hz), 2.70–2.68 (d, 2H, *J* = 7.5 Hz), 2.64 (s, 3H), 2.62–2.61 (d, 1H, *J* = 4.1 Hz), 2.55 (br s, 4H), 2.52 (s, 1H), 2.48 (s, 3H), 2.39 (s, 3H), 2.36–2.35 (d, 2H, *J* = 6.9 Hz), 2.33 (s,1H), 2.26 (s,1H), 1.87–1.85 (d, 2H, *J* = 11.0 Hz), 1.50 (s, 1H), 1.35–1.33 (t, 3H, *J* = 7.1 Hz). ^{13}C NMR (125 MHz, CD_3OD): 184.1, 180.4, 169.9, 165.8, 138.4, 133.2, 132.8, 129.9, 116.1, 115.4, 108.5, 104.5, 67.6, 65.9, 62.5, 60.3, 56.9, 56.1, 55.6, 55.0, 55.0, 54.8, 31.3, 28.6, 16.2, 14.8. HRMS (*m/z*): [MH+] calcd for $C_{26}H_{39}N_4O_4$, 471.2966; found 471.2982.

(*E*)-Ethyl 5-((2,5-dimethyl-1-(1-(2-(piperidin-1-yl)ethyl)piperidin-4-yl)-1*H*-pyrrol-3-yl)methylene)-2-methyl-4-oxo-4,5-dihydro-1*H*-pyrrole-3-carboxylate (38).

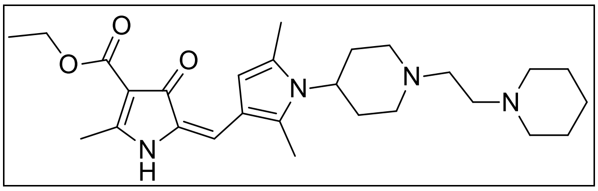

Yellow powder (0.011 g, 57%), mp 122–125 ºC. ^{1}H NMR (500 MHz, CD_3OD): *δ* 7.13 (s, 1H) , 6.50 (s, 1H), 4.23–4.16 (m, 2H), 4.12–4.06 (m, 1H), 3.73–3.71 (m, 4H), 3.23–3.22 (d, 2H, *J* = 8.1 Hz), 2.70–2.68 (d, 2H, *J* = 7.5 Hz), 2.64 (s, 3H), 2.62–2.61 (d, 1H, *J* = 4.1 Hz), 2.55 (br s, 4H), 2.52 (s, 1H), 2.48 (s, 3H), 2.39 (s, 3H), 2.36–2.35 (d, 2H, *J* = 6.9 Hz), 2.33 (s, 1H), 2.26 (s, 2H), 1.87-1.85 (d, 2H, *J* = 11.0 Hz), 1.52 (s, 1H), 1.34-1.32 (t, 3H, *J* = 7.1 Hz). ^{13}C NMR (125 MHz, CD_3OD): 184.1,169.9, 165.8, 138.4, 131.6, 131.5, 128.6, 116.1, 115.4, 108.5, 104.5, 67.3, 61.1, 58.1, 52.5, 38.0, 29.7, 28.3, 23.2, 22.6, 22.3, 21.6, 15.7, 15.0, 14.4, 13.8, 10.7. HRMS (*m/z*): [MH+] calcd for $C_{27}H_{41}N_4O_3$, 469.3173; found: 469.3187.

(*E*)-Ethyl 5-((1-(1-(cyclopropylmethyl)piperidin-4-yl)-2,5-dimethyl-1*H*-pyrrol-3-yl)methylene)-2-methyl-4-oxo-4,5-dihydro-1*H*-pyrrole-3-carboxylate (39).

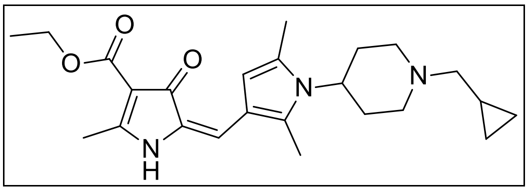

Yellow powder (0.065 g, 77%), mp 142–146 ºC. ^{1}H NMR (500 MHz, DMSO-d_6): *δ* 9.45 (s, 1H, -N*H*), 6.88 (s, 1H), 6.30 (s, 1H), 6.01 (s, 1H), 4.04–4.01 (m, 3H), 3.44–3.39 (m, 2H), 2.65 (s, 2H), 2.42 (s, 2H), 2.39 (s, 3H), 2.26 (s, 3H), 2.17 (s, 2H), 2.12 (s, 3H), 1.83–1.72 (d, 2H, *J* = 2.9, 12.8 Hz), 1.13–1.16 (t, 3H, *J* = 14.3 Hz), 0.97–0.94 (m, 2H), 0.50–0.49 (q, 2H, *J* = 4.9 Hz). ^{13}C NMR (125 MHz, DMSO-d_6): 187.2, 184.1, 170.1, 165.7, 164.8, 132.8, 132.0, 130.1, 115.8, 104.4,64.0, 63.3, 61.1, 60.3, 58.3, 54.3, 54.1, 53.6, 29.9, 25.2, 18.3, 16.3, 15.0, 4.4. HRMS (*m/z*): [MH+] calcd for $C_{24}H_{34}N_3O_3$, 412.2595; found: 412.2588.

(*E*)-Ethyl 5-((1-(1-(cyclohexylmethyl)piperidin-4-yl)-2,5-dimethyl-1*H*-pyrrol-3-yl)methylene)-2-methyl-4-oxo-4,5-dihydro-1*H*-pyrrole-3-carboxylate (40).

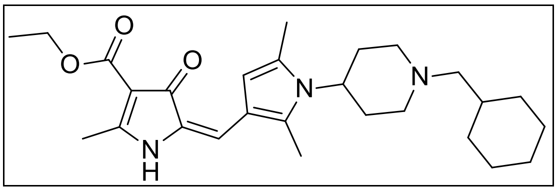

Yellow powder (0.02 g, 29%), mp 184–186 ºC. ^{1}H NMR (500 MHz, CD_3OD): δ 10.09 (s, 1H, -N*H*), 6.64 (s, 1H), 6.50 (s, 1H), 4.22–4.19 (m, 1H), 4.12–4.09 (t, 2H, *J* = 7.1 Hz), 4.08–3.98 (m, 2H), 3.85–3.84 (dd, 1H, *J* = 2.7, 2.8 Hz), 3.83–3.81 (m, 2H), 3.31 (s, 3H), 3.29 (d, 1H, J = 1.4 Hz), 3.27 (d, 1H, *J* = 1.6 Hz), 2.97-2.94 (d, 2H, *J* = 11.1 Hz), 2.41 (q, 1H, *J* = 1.6 Hz), 2.36 (s, 3H), 2.31 (s, 3H), 2.19–2.17 (td, 2H, *J* = 7.2 Hz), 2.14–2.11 (td, 1H, *J* = 3.1, 9.1 Hz), 2.10–2.09 (td, 2H, *J* = 3.1, 9.1 Hz), 2.07–2.02 (t, 2H, *J* = 3.1, 9.1 Hz), 1.74–1.72 (dd, 2H, *J* = 11.1 Hz), 1.64–1.61 (dd, 2H, *J* = 11.1 Hz), 1.23–1.20 (m, 3H). HRMS (*m/z*): [MH+] calcd for $C_{27}H_{40}N_3O_3$, 454.3064; found 454.3053.

(*E*)-Ethyl 5-((2,5-dimethyl-1-(1-((tetrahydro-2*H*-pyran-4-yl)methyl)piperidin-4-yl)-1*H*-pyrrol-3-yl)methylene)-2-methyl-4-oxo-4,5-dihydro-1*H*-pyrrole-3-carboxylate (41).

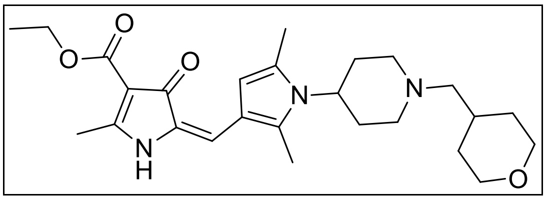

Yellow powder, (0.05 g, 66%), mp 180–183 ºC. ^{1}H NMR (500 MHz, DMSO-d_6): δ 10.09 (s, 1H, -N*H*), 6.64 (s, 1H), 6.50 (s, 1H), 4.22–4.19 (m, 1H), 4.12–4.09 (t, 2H, *J* = 7.1 Hz), 4.08–3.98 (m, 2H), 3.85–3.84 (dd, 1H, *J* = 2.7, 2.8 Hz), 3.83–3.81 (m, 2H), 3.31 (s, 3H), 3.29 (d, 1H, *J* = 1.4 Hz), 3.27 (d, 1H, *J* = 1.6 Hz), 2.97-2.94 (d, 2H, *J* = 11.1 Hz), 2.41 (q, 1H, *J* = 1.6 Hz), 2.36 (s, 3H), 2.31 (s, 3H), 2.19–2.17 (td, 2H, *J* = 7.2 Hz), 2.14–2.11 (td, 1H, *J* = 3.1, 9.1 Hz), 2.07–2.02 (t, 2H, *J* = 3.1, 9.1 Hz), 1.74–1.72 (dd, 2H, *J* = 11.1 Hz), 1.64–1.61 (dd, 2H, *J* = 11.1 Hz), 1.23–1.20 (m, 3H). ^{13}C-NMR (125 MHz, DMSO-d_6): 180.3, 169.2, 163.3, 144.9, 134.5, 130.1, 128.5, 113.0, 110.0, 107.1, 102.5, 102.4, 66.8, 63.8, 58.6, 58.0, 57.8, 54.7, 53.3, 32.1, 31.3, 30.6, 17.1, 15.7, 15.7, 14.4. HRMS (*m/z*): [MH+] calcd for $C_{26}H_{38}N_3O_4$, 456.2857; found: 456.2844.

(*E*)-Ethyl 5-((2,5-dimethyl-1-(1-((1-methylpiperidin-4-yl)methyl)piperidin-4-yl)-1*H*-pyrrol-3-yl)methylene)-2-methyl-4-oxo-4,5-dihydro-1*H*-pyrrole-3-carboxylate (42).

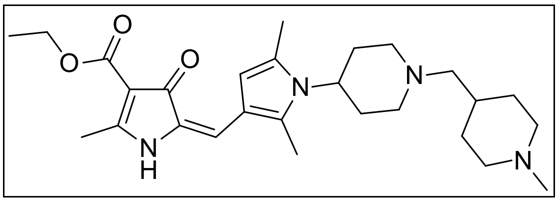

Yellow powder (0.027 g, 38%), mp 230–235 ºC. ^{1}H NMR (500 MHz, CD_3OD): *δ* 9.68 (s, 1H, -N*H*), 7.12 (s, 1H), 6.55 (s, 1H), 4.57 (s, 1H), 4.27–4.26 (m, 2H), 3.71 (br s, 2H), 3.57–3.56 (m, 2H), 3.37 (s, 1H), 3.10 (br s, 2H), 3.05 (br s, 2H), 3.02 (s, 1H), 2.89 (br s, 3H), 2.77–2.72 (m, 2H), 2.66 (s, 3H), 2.51 (s, 3H), 2.42 (s, 3H), 2.38 (s, 1H), 2.22–2.19 (d, 2H, *J* =14.2 Hz), 2.07–2.05 (d, 2H, *J* = 6.8 Hz), 1.68–1.66 (d, 2H, *J* = 9.4 Hz), 1.36–1.33 (t, 3H, *J* = 7.1 Hz). ^{13}C NMR (125 MHz, CD_3OD): 184.3, 170.9, 170.0, 165.8, 164.8, 138.5, 133.3, 130.0, 116.0, 115.6, 108.7, 104.4, 66.0, 65.6, 65.4, 65.0, 62.7, 60.4, 54.5, 53.9, 43.8, 37.0, 30.7, 29.5, 16.4, 14.8, 11.6. HRMS (*m/z*): [MH+] calcd for $C_{27}H_{41}N_4O_3$, 469.3173; found: 469.3177.

(*E*)-Ethyl 5-((2,5-dimethyl-1-(1-((6-(trifluoromethyl)pyridin-3-yl)methyl)piperidin-4-yl)-1*H*-pyrrol-3-yl)methylene)-2-methyl-4-oxo-4,5-dihydro-1*H*-pyrrole-3-carboxylate (43).

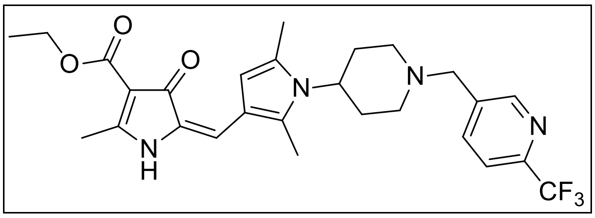

Yellow powder (0.022 g, 16%), mp 140–143 ºC. ^{1}H NMR (500 MHz, DMSO-d_6): *δ* 10.14 (s, 1H, -N*H*), 8.79–8.78 (d, 1H, *J* = 1.2 Hz), 8.11–8.09 (dd, 1H, *J* = 1.1 Hz) 8.11–8.09 (dd, 1H, J = 1.1, 6.6 Hz), 7.95–7.93 (d, 1H, *J* = 8.0 Hz), 6.70 (s, 1H), 6.56 (s, 1H), 4.17–4.14 (m, 3H), 4.12–4.09 (m, 2H), 3.75 (br s, 2H), 2.99–2.98 (m, 2H), 2.95 (s, 1H), 2.60 (s, 1H), 2.56 (m, 3H), 2.48 (s, 3H), 2.26-2.40 (m, 2H), 1.81–1.80 (m, 2H), 1.28–1.25 (m, 3H). ^{13}C NMR (125 MHz, DMSO-d_6): 180.3, 169.2, 163.6, 163.3, 150.3, 145.3, 145.0, 138.3, 138.0, 134.5, 130.1, 129.8, 128.5, 120.6, 120.4, 113.1, 110.6, 107.2, 102.4, 58.2, 58.0, 57.8, 54.4, 52.6, 30.6, 15.7, 14.4. HRMS (*m/z*): [MH+] calcd for $C_{27}H_{32}F_3N_4O_3$, 517.2421; found 517.2414.

(*E*)-Ethyl 5-((1-(1-(4-fluoro-2-(trifluoromethyl)benzyl)piperidin-4-yl)-2,5-dimethyl-1*H*-pyrrol-3-yl)methylene)-2-methyl-4-oxo-4,5-dihydro-1*H*-pyrrole-3-carboxylate (44).

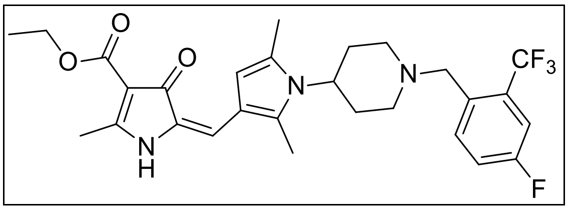

Yellow powder (0.038 g, 55%), mp 145–148 ºC. ^{1}H NMR (500 MHz, Acetone-d$_6$): δ 7.98 (m, 2H), 7.56–7.44 (m, 1H) 6.86 (s, 1H), 6.42 (s, 1H), 5.64 (s, 1H), 4.18–4.14 (m, 2H), 3.70 (br s, 2H), 2.80 (s, 3H), 2.62 (br s, 2H), 2.44 (br s, 2H), 2.31–2.29 (m, 2H), 2.17–2.06 (m, 6H), 1.84–1.82 (m, 2H), 1.27–1.24 (t, 3H, *J* = 7.0 Hz), ^{13}C NMR (125 MHz, Acetone-d$_6$): 181.8, 170.0, 164.6, 162.8, 160.9, 135.1, 134.0(2C), 131.3, 130.1, 120.0, 129.8, 114.0, 113.8, 111.5, 59.1, 58.1, 55.9, 55.0, 54.8(2C), 36.2, 31.93(2C), 31.0, 16.1, 14.9, 11.5 HRMS (*m/z*): [MH+] calcd for $C_{28}H_{32}F_4N_3O_3$, 534.2374; found 534.2352.

(*E*)-Ethyl 5-((2,5-dimethyl-1-(1-((1-methyl-1H-pyrazol-3-yl)methyl)piperidin-4-yl)-1*H*-pyrrol-3-yl)methylene)-2-methyl-4-oxo-4,5-dihydro-1*H*-pyrrole-3-carboxylate (45).

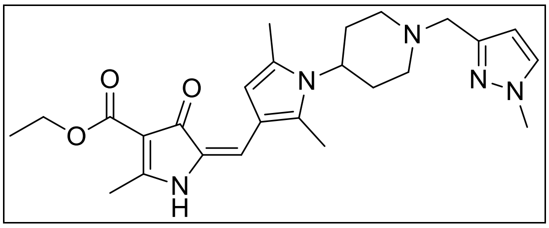

Yellow powder (0.015 g, 40%), mp 180–184 ºC. ^{1}H NMR (500 MHz, CD_3CN): δ 9.75 (s, 1H, -N*H*), 7.43 (d, 1H, *J* = 2.0 Hz), 6.80 (s, 1H), 6.31 (s, 1H), 6.18–6.17 (d, 1H, *J* = 2.1 Hz), 4.20–4.16 (m, 2H), 4.07–4.02 (q, 1H, *J* = 7.1 Hz), 3.82 (s, 2H), 3.54 (d, 2H, *J* = 2.9 Hz), 3.06–3.04 (d, 2H, *J* = 8.9 Hz), 2.59 (s, 3H), 2.36 (s, 3H), 2.29 (d, 3H, *J* = 2.8 Hz), 2.24–2.23 (d, 2H, *J* =2.9 Hz), 2.21–2.18 (d, 3H, *J* = 7.5 Hz), 1.77–1.75 (d, 2H, *J* = 11.6 Hz), 1.29–1.26 (m, 3H) ^{13}C NMR (125 MHz, CD_3CN): 180.9, 168.9, 163.5, 148.4, 148.2, 134.8, 130.6, 130.5, 130.0, 128.8, 113.2, 110.5, 106.8, 105.0, 103.0, 58.3, 54.8, 54.7, 52.5, 37.7, 30.3, 15.1, 13.6(2C), 10.4. HRMS (*m/z*): [MH+] calcd for $C_{25}H_{34}N_5O_3$, 452.2656; found 452.2647.

(*E*)-Ethyl 5-((1-(1-((1H-imidazol-2-yl)methyl)piperidin-4-yl)-2,5-dimethyl-1*H*-pyrrol-3-yl)methylene)-2-methyl-4-oxo-4,5-dihydro-1*H*-pyrrole-3-carboxylate (46).

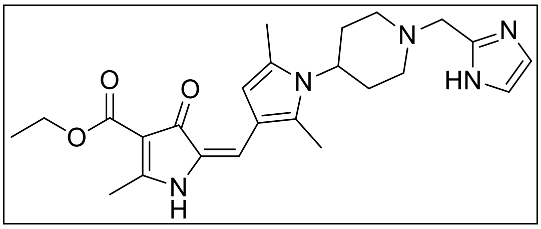

Yellow powder (0.045 g, 99%), mp 185–190 ºC. ^{1}H NMR (500 MHz, DMSO-d$_6$): δ 10.09 (s, 1H, -N*H*), 6.93 (br s, 1H), 6.64 (s, 1H), 6.50 (s, 1H), 4.12–4.08 (m, 3H), 4.05–4.00 (m, 2H), 3.56 (s, 2H), 2.94–2.92 (d, 2H, *J* = 6.8 Hz), 2.55 (s, 3H), 2.37 (s, 3H), 2.31 (s, 3H), 2.18–2.12 (m, 4H), 1.73 (br s, 2H), 1.23–1.20 (m, 3H). ^{13}C NMR (125 MHz, DMSO-d$_6$): 180.3, 169.2, 163.3, 144.4, 134.5, 130.0, 128.5, 117.3, 113.1, 110.0, 102.4, 58.0, 54.7, 54.5, 52.7, 30.4, 15.7, 14.4, 11.0. HRMS (*m/z*): [MH+] calcd for $C_{24}H_{32}N_5O_3$, 438.2500; found 438.2489.

(*E*)-Ethyl 5-((2,5-dimethyl-1-(1-((5-methylisoxazol-3-yl)methyl)piperidin-4-yl)-1*H*-pyrrol-3-yl)methylene)-2-methyl-4-oxo-4,5-dihydro-1*H*-pyrrole-3-carboxylate (47).

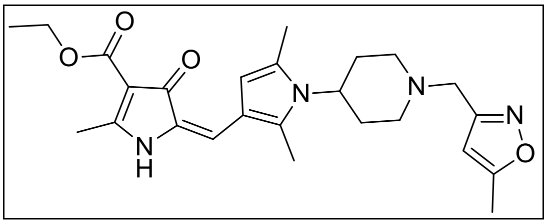

Yellow powder (0.050 g, 33%), mp: 185–188 ºC. ^{1}H NMR (500 MHz, DMSO-d$_6$): δ 10.09 (s, 1H, -N*H*), 6.64 (s, 1H), 6.50 (s, 1H), 6.22 (d, 1H), 4.11–4.07 (m, 2H), 4.04–3.99 (m, 2H), 3.56 (s, 2H) 3.31 (s, 3H), 2.94–2.92 (d, 2H, *J* = 8.6 Hz), 2.55 (s, 3H), 2.40 (d, 1H, *J* = 0.6 Hz), 2.37 (s, 3H), 2.31 (s, 3H), 2.21–2.22 (m, 2H), 1.76–1.74 (d, 2H, *J* = 11.3 Hz), 1.23–1.20 (m, 3H). ^{13}C NMR (125 MHz; DMSO-d$_6$): 180.3, 178.8, 169.2, 169.2, 163.3, 161.0, 134.5, 130.1, 129.5, 128.5, 113.1, 110.0, 107.2, 102.4, 102.0, 58.0, 54.4, 52.6, 52.4, 52.1, 30.5, 15.7, 14.0, 11.7, 11.0. HRMS (*m/z*): [MH+] calcd for $C_{25}H_{33}N_4O_4$, 453.2496; found 453.2512.

(*E*)-Ethyl 5-((2,5-dimethyl-1-(1-(oxazol-4-ylmethyl)piperidin-4-yl)-1*H*-pyrrol-3-yl)methylene)-2-methyl-4-oxo-4,5-dihydro-1*H*-pyrrole-3-carboxylate (48).

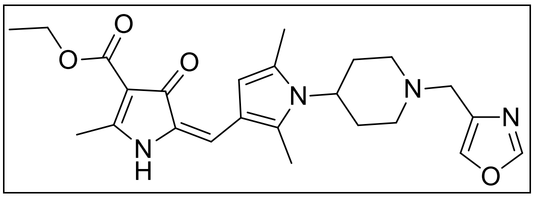

Yellow powder (0.03 g, 78%), mp 180–182 ºC. ^{1}H NMR (500 MHz, DMSO-d$_6$): δ 10.14 (s, 1H, -N*H*), 8.37 (d, 1H, *J* = 0.5 Hz), 8.07 (d, 1H, *J* =0.5 Hz), 6.69 (s, 1H), 6.55 (s, 1H), 4.48 (m, 1H), 4.17–4.12 (m, 2H), 4.08 –4.05 (m, 2H), 3.54 (s, 2H) 3.05–3.02 (d, 2H, *J* = 7.6 Hz), 2.60 (s, 3H), 2.42 (s, 3H), 2.36 (s, 3H), 2.23–2.21 (d, 2H, *J* = 9.2 Hz), 1.80–1.78 (d, 2H, *J* = 8.8 Hz), 1.28–1.24 (m, 3H), ^{13}C NMR (125 MHz, DMSO-d$_6$): 180.3, 178.2, 169.2, 163.3, 151.8, 137.1, 136.1, 130.1, 129.8, 128.5, 113.0, 110.0, 102.5, 102.4, 58.0, 54., 52.5, 58.0, 52.5, 52.4, 30.5, 15.7, 12.5, 14.4. HRMS (*m/z*): [MH+] calcd for $C_{24}H_{31}N_4O_4$, 439.2340; found 439.2336.

(*E*)-Ethyl 5-((2,5-dimethyl-1-(1-((1-methyl-1*H*-imidazol-2-yl)methyl)piperidin-4-yl)-1*H*-pyrrol-3-yl)methylene)-2-methyl-4-oxo-4,5-dihydro-1*H*-pyrrole-3-carboxylate (49).

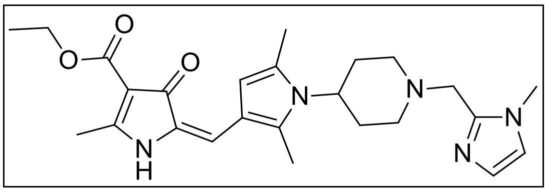

Yellow powder (0.056 g, 74%), mp 186–189ºC. ^{1}H NMR (500 MHz, DMSO-d$_6$): δ 10.09 (s, 1H, -N*H*), 7.09–7.08 (d, 1H, *J* = 1.0 Hz), 6.76 (d, 1H, *J* =1.1Hz), 6.64 (s, 1H), 6.50 (s, 1H), 4.22–4.19 (m, 1H), 4.12–4.09 (q, 2H, *J* = 7.1 Hz), 4.06–4.00 (m, 2H), 3.57 (s, 2H) 3.32 (s, 3H), 2.91–2.89 (d, 2H, *J* = 9.4 Hz), 2.55 (s, 3H), 2.37 (s, 3H), 2.34 (s, 3H), 2.19–2.10 (td, 2H, *J* = 9.4, 9.2, 2.6 Hz), 1.75–1.73 (d, 2H, *J* = 1.4, 7.6 Hz), 1.23–1.20 (m, 3H). ^{13}C NMR (125 MHz; DMSO-d$_6$): 180.3, 169.2, 163.3, 144.3, 134.4, 130.0, 128.5, 126.0, 121.9, 121.9, 113.1, 110.0, 107.2, 102.4, 58.0, 57.8, 54.5, 54.5, 54.3, 52.7, 32.4, 30.5, 15.7, 14.4, 11.0. HRMS (*m/z*): [MH+] calcd for $C_{25}H_{34}N_5O_3$, 452.2656; found: 452.2659.

(*E*)-Ethyl 5-((2,5-dimethyl-1-(1-((1-methyl-1*H*-pyrrol-2-yl)methyl)piperidin-4-yl)-1*H*-pyrrol-3-yl)methylene)-2-methyl-4-oxo-4,5-dihydro-1*H*-pyrrole-3-carboxylate (50).

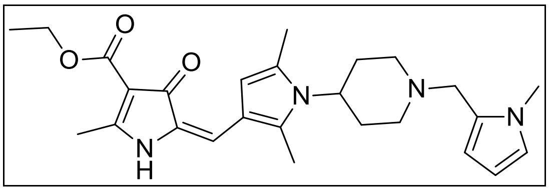

Yellow powder (0.07 g, 44%), mp 190–194 ºC. ^{1}H NMR (500 MHz, DMSO-d$_6$): *δ* 10.09 (s, 1H, -N*H*), 6.80–6.67 (t, 1H, *J* = 1.8 Hz), 6.64 (s, 1H), 6.50 (s, 1H), 5.89–5.87 (m, 2H), 4.22–4.19 (m, 1H), 4.12–4.11 (q, 2H, *J* = 7.1 Hz), 4.08–4.09 (q, 2H, *J* = 7.1 Hz), 3.81 (m, 1H), 3.62–3.59 (m, 3H), 2.97–2.95 (d, 1H, *J* = 9.2 Hz), 2.55 (s, 3H), 2.41 (q, 2H, *J* = 1.5 Hz), 2.37 (s, 3H), 2.31 (s, 3H), 2.11–2.05 (m, 2H), 1.76–1.74 (d, 2H, J = 11.0 Hz), 1.21–1.19 (m, 3H). ^{13}C NMR (125 MHz, DMSO-d$_6$): 180.3, 169.3, 163.3, 160.8, 144.9, 134.5, 130.4, 130.1, 128.6, 128.5, 122.7, 113.1, 110.0, 105.9, 102.4, 58.6, 57.8, 54.8, 54.3, 52.9, 52.3, 33.4, 30.3, 17.0, 14.4, 11.1. HRMS (*m/z*): [MH+] calcd for $C_{26}H_{35}N_4O_3$, 451.2704; found 451.2699.

(*E*)-Ethyl 5-((2,5-dimethyl-1-(1-(4-(5-methyl-1,2,4-oxadiazol-3-yl)benzyl)piperidin-4-yl)-1*H*-pyrrol-3-yl)methylene)-2-methyl-4-oxo-4,5-dihydro-1*H*-pyrrole-3-carboxylate (51).

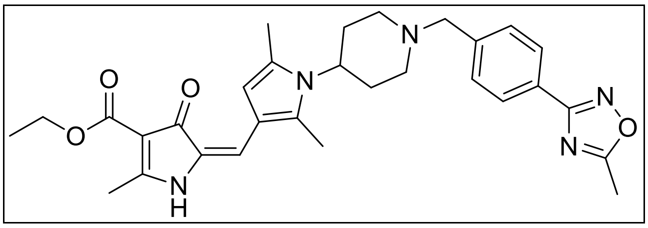

Yellow powder (0.018 g, 26%), mp 195–200 ºC. ^{1}H NMR (500 MHz, DMSO-d$_6$): *δ* 10.15 (s, 1H, -N*H*), 8.08–8.07 (d, 2H, *J* = 7.5 Hz), 7.75–7.74 (d, 2H, *J* = 7.5 Hz), 6.63 (s, 1H), 6.54 (s, 1H), 4.13–4.05 (m, 4H), 4.04–4.02 (q, 1H, *J* = 7.1 Hz), 3.01 (br s, 2H), 2.69 (br s, 2H), 2.55 (s, 3H), 2.48 (s, 3H), 2.39 (s, 3H), 2.33 (s, 3H), 2.24–2.23 (d, 2H, *J* =2.9 Hz),1.95–1.93 (d, 2H, *J* = 10.9 Hz), 1.22–1.19 (t, 3H, *J* = 7.0 Hz). ^{13}C NMR (125 MHz, DMSO-d$_6$): 180.3, 177.6, 169.4, 167.2, 163.3, 134.6, 132.2, 132.1, 131.8, 131.2, 130.1(2C), 128.6(2C), 127.2, 126.9, 113.2, 109.9, 107.3, 102.3, 58.1, 51.8, 51.3, 28.8, 27.9, 15.7, 15.0, 14.4, 14.0, 12.03. HRMS m/z calculated for $C_{30}H_{36}N_5O_4$, 530.2762; found 530.2762.

(*E*)-Ethyl 5-((2,5-dimethyl-1-(1-((6-morpholinopyridin-3-yl)methyl)piperidin-4-yl)-1*H*-pyrrol-3-yl)methylene)-2-methyl-4-oxo-4,5-dihydro-1*H*-pyrrole-3-carboxylate (52).

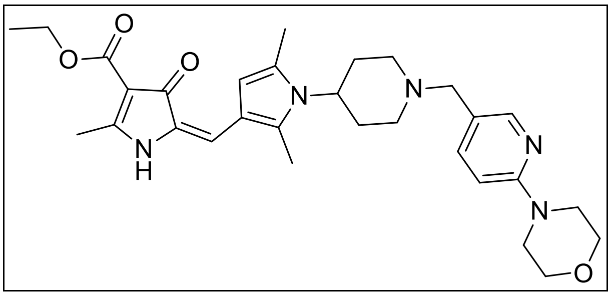

Yellow powder (0.07 g, 85%), mp >280 ºC. ^{1}H NMR (500 MHz, DMSO-d$_6$): δ 10.30 (s, 1H, -N*H*), 8.30 (s, 1H), 8.05 (s, 1H), 7.15 (s, 1H), 6.72 (s, 1H) , 6.61 (s, 1H), 4.28 (m, 1H), 4.25–4.28 (m, 2H), 4.11–4.10 (d, 2H, *J* = 6.95 Hz), 3.80 (s, 2H), 3.72 (s, 3H), 3.45–3.43 (d, 2H, *J* = 9.55 Hz), 3.10–3.08 (d, 2H, *J* = 9.55 Hz), 2.69–2.67 (m, 2H), 2.57 (s, 2H), 2.51 (s, 4H), 2.41 (s, 3H), 2.35 (s, 3H), 1.97 (d, 2H, *J* =11.5 Hz), 1.21 (t, 3H, *J* = 7.10 Hz). ^{13}C NMR (125 MHz, DMSO-d$_6$): 180.1, 179.6, 169.1, 163.2, 163.2, 130.56 (3C), 128.24(2C), 114.2, 111.3, 102.4, 65.66(2C), 65.5, 58.22 (2C), 54.9, 54.9, 51.4, 50.28(2C), 58.2, 50.2, 45.4, 27.1, 15.7, 14.4, 14.19. HRMS m/z calculated for $C_{30}H_{40}N_5O_4$, 534.3075; found 534.3057.

(*E*)-Ethyl 5-((2,5-dimethyl-1-(1-((2-morpholinopyrimidin-5-yl)methyl)piperidin-4-yl)-1*H*-pyrrol-3-yl)methylene)-2-methyl-4-oxo-4,5-dihydro-1*H*-pyrrole-3-carboxylate (53).

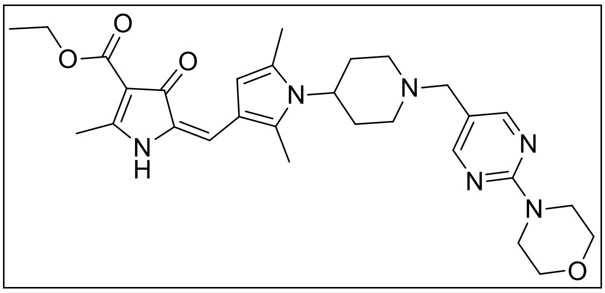

Yellow powder (0.22 g, 89%), mp 194–199 ºC. ^{1}H NMR (500 MHz, DMSO-d$_6$): δ 10.18 (s, 1H, -N*H*), 8.33 (s, 2H), 6.63 (s, 1H), 6.50 (s, 1H), 4.43 (s, 1H) , 4.12–4.07 (m, 3H, *J* = 4.8, 2.2, 7.1 Hz), 4.02 (br s, 2H,), 3.44–3.42 (d, 2H, *J* = 7.0 Hz), 3.36 (s, 3H), 2.95–2.93 (d, 2H, *J* = 6.0 Hz), 2.56–2.55 (d, 2H, *J* = 5.4 Hz), 2.51–2.50 (q, 3H, *J* = 1.7 Hz), 2.43–2.41 (d, 1H, *J* = 8.0 Hz), 2.36 (s, 2H), 2.31 (s, 2H), 2.18–2.10 (m, 3H), 1.75–1.74 (d, 2H, *J* = 8.3 Hz), 1.22–1.19 (t, 2H, *J* = 7.1 Hz), 1.13–1.10 (t, 3H, *J* = 6.9 Hz). ^{13}C NMR (125 MHz, DMSO-d$_6$): 180.4, 169.8, 169.2, 163.3, 158.5, 134.6, 130.1, 129.8, 128.8, 128.5, 118.8, 113.0, 110.0, 102.4, 65.9, 63.9, 58.1, 58.0, 57.8, 55.8, 54.4, 52.2, 52.0, 43.9, 30.4, 15.7, 15.0, 14.4, 11.0. HRMS m/z calculated for $C_{29}H_{39}N_6O_4$, 535.3027; found 535.3050.

(*E*)-Ethyl 5-((2,5-dimethyl-1-(1-(4-morpholinobenzyl)piperidin-4-yl)-1*H*-pyrrol-3-yl)methylene)-2-methyl-4-oxo-4,5-dihydro-1*H*-pyrrole-3-carboxylate (54).

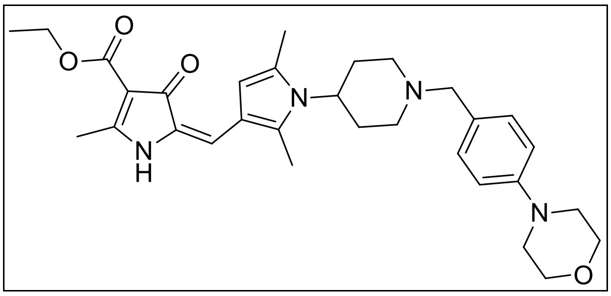

Yellow powder (0.07 g, 85%), mp >280 ºC. ^{1}H NMR (500 MHz, DMSO-d$_6$): δ 10.30 (s, 1H, -N*H*), 8.24-8.22 (d, 2H, *J* = 8.45 Hz), 7.99 -7.97 (d, 2H, *J* = 8.6, 8.45 Hz), 6.51 (s, 1H) , 6.41 (s, 1H), 4.28 (m, 1H), 4.21–4.24 (m, 2H), 4.11–4.10 (d, 2H, *J* = 6.95 Hz), 3.80 (s, 2H), (3.72 (s, 3H), 3.49–3.45 (d, 2H, *J* = 9.55 Hz), 3.10–3.08 (d, 2H, *J* = 9.55 Hz), 2.69–2.67 (m, 2H), 2.54 (s, 2H), 2.51 (s, 4H), 2.40 (s, 3H), 2.35 (s, 3H), 1.94 (d, 2H, *J* =11.5 Hz), 1.21 (t, 3H, *J* = 7.10 Hz). ^{13}C NMR (125 MHz, DMSO-d$_6$): 180.2, 178.9, 166.8, 163.4, 163.0, 129.9 (3C), 128.0 (2C), 114.0, 111.9, 101.9, 65.9, 65.6 (2C), 65.5, 58.22 (2C), 54.9, 54.9, 51.4, 51.08 (2C), 58.1, 50.0, 45.2, 27.8, 15.3, 14.7, 14.1. HRMS m/z calculated for $C_{31}H_{41}N_4O_4$, 533.0035; found 533.4513.

General procedure for the synthesis of pyrrolones(56-64).

A mixture of enaminoester **55** (1.0 equiv) and primary amine (4.0 equiv) in *iso*-propanol was heated at reflux for 6 h or irradiated by microwave for 15–30 min at 110 ºC and cooled. The precipitate was filtered and washed with ice-cooled methanol or ethanol to give the required product.

(*E*)-2-((2,5-Dimethyl-1-(2-(trifluoromethyl)phenyl)-1H-pyrrol-3-yl)methylene)-1*H*-pyrrolo[3,2-c]pyridine-3,4(2*H*,5*H*)-dione (56).

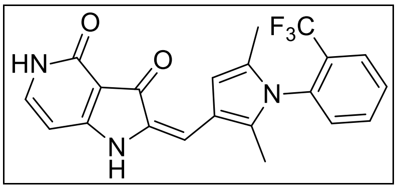

Yellow powder (0.22 g, 89%), mp 185–190 ºC. ^{1}H NMR (500 MHz, DMSO-d$_6$): δ 10.92 (s, 1H, -N*H*), 10.18 (s, 1H, -N*H*), 8.02–8.01 (d, 1H, *J* = 13.3 Hz), 7.93–7.90 (t, 1H, *J* = 6.9 Hz), 7.83 –7.80 (t, 1H, *J* = 7.4 Hz), 7.53–7.51 (d, 1H, *J* = 7.7 Hz), 7.45–7.43 (q, 1H, *J* = 7.0 Hz), 6.62 (br s, 2H), 6.19–6.18 (d, 1H, *J* = 6.9 Hz), 1.99 (s, 3H), 1.92 (s, 3H). ^{13}C NMR (125

MHz, DMSO-d_6): 180.5, 161.9, 158.2, 141.5, 134.6, 134.3, 131.7, 131.3, 130.6, 130.3, 129.1, 127.4, 124.0, 114.6, 106.9, 105.6, 103.9, 93.7, 92.9, 12.0, 10.3. HRMS m/z calculated for $C_{21}H_{17}F_3N_3O_2$, 400.1267; found $[M+H]^+$ 400.1276.

(*E*)-2-((2,5-Dimethyl-1-(2-(trifluoromethyl)phenyl)-1*H*-pyrrol-3-yl)methylene)-5-methyl-1*H*-pyrrolo[3,2-c]pyridine-3,4(2*H*,5*H*)-dione (57).

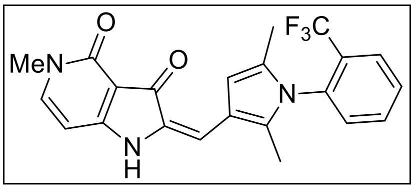

Yellow powder (0.22 g, 89%), mp 194–198 ºC. ^{1}H NMR (500 MHz, DMSO-d_6): δ 9.94 (s, 1H, -N*H*), 8.02–8.01 (d, 1H, *J* = 7.2 Hz), 7.94–7.91 (t, 1H, *J* = 7.0 Hz), 7.83–7.80 (t, 1H, *J* = 7.5 Hz), 7.78–7.76 (d, 1H, *J* = 7.2 Hz), 7.53–7.51 (d, 1H, *J* = 7.7 Hz), 6.63 (br s, 2H), 6.21–6.20 (d, 1H, *J* = 7.2 Hz), 2.00 (s, 3H), 1.92 (s, 3H), 0.88–0.82 (t, 3H, *J* = 7.25Hz). ^{13}C NMR (125 MHz, DMSO-d_6): 180.5, 179.0, 161.1, 157.3, 145.6, 134.7, 134.3, 131.7, 131.3, 130.8, 130.3, 116.0, 114.0, 108.7, 106.9, 105.6, 103.4, 93.5, 92.7, 35.4, 12.0, 10.3. HRMS m/z calculated for $C_{22}H_{19}F_3N_3O_2$, 414.1424; found $[M+H]^+$ 414.1418.

(*E*)-2-((2,5-Dimethyl-1-(2-(trifluoromethyl)phenyl)-1*H*-pyrrol-3-yl)methylene)-5-ethyl-1*H*-pyrrolo[3,2-c]pyridine-3,4(2*H*,5*H*)-dione (58).

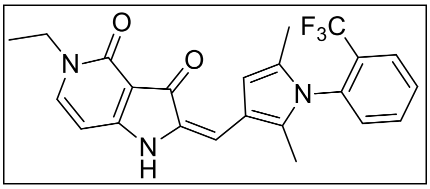

Yellow powder (0.21 g, 89%), mp 204–205 ºC. ^{1}H NMR (500 MHz, DMSO-d_6): δ 9.99 (s, 1H, -N*H*), 8.08–8.06 (d, 1H, *J* = 7.0 Hz), 7.99–7.96 (t, 1H, *J* = 6.7, 7.6 Hz), 7.89–7.86 (t, 1H, *J* = 7.6 Hz), 7.85–7.83 (t, 1H, *J* = 7.2 Hz), 7.58–7.57 (d, 1H, *J* = 7.7 Hz), 6.67 (br s , 2H), 6.28–6.26 (d, 1H, *J* = 7.2 Hz), 3.92–3.87 (m, 2H), 2.05 (s, 3H), 1.97 (s, 3H), 1.26–1.23 (m, 3H). ^{13}C NMR (125 MHz, DMSO-d_6): 180.6, 161.0, 156.7, 144.6, 135.0, 134.7, 134.3, 131.7, 131.3, 130.8, 130.3, 129.1, 116.0, 114.0, 108.7, 106.9, 105.6, 103.6, 93.0, 42.2, 15.0, 12.0, 10.3. HRMS m/z calculated for $C_{23}H_{21}F_3N_3O_2$, 428.1580; found $[M+H]^+$ 428.1583.

(*E*)-2-((2,5-Dimethyl-1-(2-(trifluoromethyl)phenyl)-1*H*-pyrrol-3-yl)methylene)-5-propyl-1*H*-pyrrolo[3,2-c]pyridine-3,4(2*H*,5*H*)-dione (59).

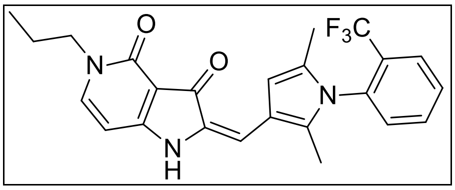

Yellow powder (0.24 g, 89%), mp 180–185 ºC. ^{1}H NMR (500 MHz, DMSO-d_6): δ 9.96 (s, 1H, -N*H*), 8.02–8.01 (d, 1H, *J* = 6.2 Hz), 7.93–7.92 (t, 1H, *J* = 7.0 Hz), 7.83–7.80 (t, 1H, *J* = 7.5 Hz), 7.77–7.76 (d, 1H, *J* = 7.2 Hz), 7.53–7.52 (d, 1H, *J* = 7.7 Hz), 6.63 (br s, 2H), 6.22–6.21 (d, 1H, *J* = 7.2 Hz), 3.78–3.74 (m, 4H), 1.98 (s, 3H), 1.91 (s, 3H), 1.64–1.59 (m, 3H). HRMS m/z calculated for $C_{24}H_{23}F_3N_3O_2$, 442.1737; found $[M+H]^+$ 442.1744.

(*E*)-2-((2,5-Dimethyl-1-(2-(trifluoromethyl)phenyl)-1*H*-pyrrol-3-yl)methylene)-5-(2-morpholinoethyl)-1*H*-pyrrolo[3,2-c]pyridine-3,4(2*H*,5*H*)-dione (60).

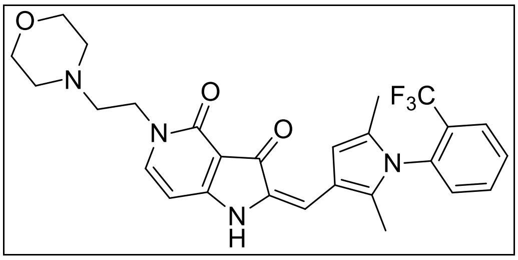

Yellow powder (0.02 g, 33%), mp 180–185 ºC. ^{1}H NMR (500 MHz, DMSO-d$_6$): δ 9.78 (s, 1H, -N*H*), 7.85–7.84 (d, 1H, *J* = 6.7 Hz), 7.77–7.74 (t, 1H, *J* = 7.1 Hz), 7.66–7.63 (t, 1H, *J* = 7.6 Hz), 7.56–7.55 (d, 1H, *J* = 8.3 Hz), 7.36–7.35 (d, 1H, *J* = 7.7 Hz), 6.46 (br s, 1H), 6.04–6.03 (d, 1H, *J* = 7.2 Hz), 5.60 (s, 1H), 3.77–3.72 (m, 4H), 2.42 (s, 1H), 1.97 (br s, 2H), 1.83 (s, 3H), 1.81 (s, 2H), 1.75 (s, 3H), 1.71 (s, 1H), 1.68 (s, 1H), 1.66 (s, 1H). HRMS m/z calculated for $C_{27}H_{28}F_3N_4O_3$, 513.2108; found $[M+H]^+$ 513.2106.

(*E*)-2-((2,5-Dimethyl-1-(2-(trifluoromethyl)phenyl)-1*H*-pyrrol-3-yl)methylene)-5-(2-(dimethylamino)ethyl)-1*H*-pyrrolo[3,2-c]pyridine-3,4(2*H*,5*H*)-dione (61).

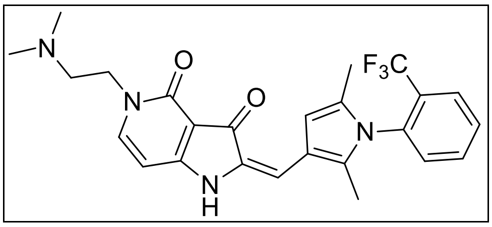

Yellow powder (0.030 g, 41%), mp 125–128 ºC. ^{1}H NMR (500 MHz, DMSO-d$_6$): δ 10.00 (s, 1H, -N*H*), 8.07–8.06 (d, 1H, *J* = 6.9 Hz), 7.99–7.96 (t, 1H, *J* = 6.8, 7.2 Hz), 7.88–7.86 (t, 1H, *J* = 7.6 Hz), 7.77–7.75 (d, 1H, *J* = 7.2 Hz), 7.58–7.57 (d, 1H, *J* = 7.7 Hz), 6.68–6.67 (d, 2H, *J* = 5.7 Hz), 6.26 -6.25 (d, 1H, *J* = 7.2 Hz), 3.99–3.91 (m, 4H), 2.23 (br s, 6H), 2.05 (s, 3H), 1.97 (s, 3H). ^{13}C NMR (125 MHz, DMSO-d$_6$): 180.6, 161.0, 157.7, 156.8, 145.5, 134.7, 134.3, 131.7, 131.3, 130.8, 130.3, 114.0, 108.7, 106.9, 105.6, 103.4, 93.3, 92.5, 58.0, 57.9, 45.2, 44.6, 44.5, 12.0, 10.3. HRMS m/z calculated for $C_{25}H_{26}F_3N_4O_2$, 471.2009; found: $[M+H]^+$ 471.2004.

(*E*)-5-Benzyl-2-((2,5-dimethyl-1-(2-(trifluoromethyl)phenyl)-1*H*-pyrrol-3-yl)methylene)-1*H*-pyrrolo[3,2-c]pyridine-3,4(2*H*,5*H*)-dione (62).

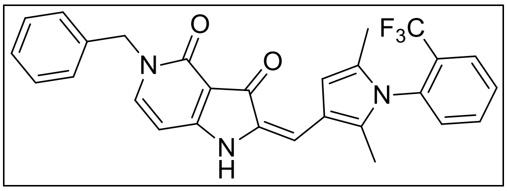

Yellow powder (0.22 g, 89%), mp 195–200 ºC. ^{1}H NMR (500 MHz, DMSO-d$_6$): *δ* 9.45 (s, 1H, -N*H*), 8.01–7.99 (d, 1H, *J* = 7.8 Hz), 7.94–7.91 (t, 2H, *J* = 7.6 Hz), 7.84–7.82 (t, 1H, *J* = 7.2 Hz), 7.69–7.67 (d, 2H, *J* = 8.2 Hz), 7.60–7.58 (d, 2H, *J* = 8.2 Hz), 7.50–7.48 (d, 1H, *J* = 7.8 Hz), 6.83 (br s, 2H), 6.54 (s, 1H), 6.39–6.37 (d, 1H, *J* = 7.2 Hz), 5.27–5.26 (m, 2H), 1.82 (s, 6H). ^{13}C NMR (125 MHz, Acetone-d$_4$): 181.7, 170.8, 162.3, 161.0, 158.2, 145.2, 143.6, 136.0, 134.8, 132.6, 132.30 (2C), 131.03(2C), 130.8, 130.3, 129.3, 129.2, 128.3, 126.3, 126.2, 115.6, 108.7, 106.5, 95.6, 50.6, 12.3, 10.7. HRMS m/z calculated for $C_{28}H_{23}F_3N_3O_2$, 490.1743; found [M+H]$^+$ 490.1752.

(*E*)-2-((2,5-Dimethyl-1-(2-(trifluoromethyl)phenyl)-1*H*-pyrrol-3-yl)methylene)-5-(4-(trifluoromethyl)benzyl)-1*H*-pyrrolo[3,2-c]pyridine-3,4(2*H*,5*H*)-dione (63).

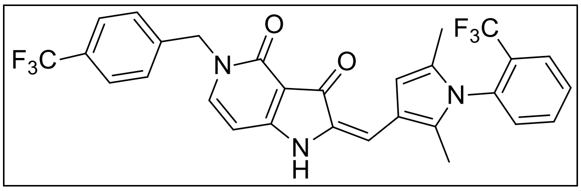

Yellow powder (0.019 g, 89%), mp 235–240 ºC. ^{1}H NMR (500 MHz, Acetone-d$_6$): *δ* 9.45 (s, 1H, -N*H*), 8.01–7.99 (d, 1H, *J* = 7.8 Hz), 7.94–7.91 (t, 2H, *J* = 7.6 Hz), 7.84–7.82 (t, 1H, *J* = 7.2 Hz), 7.69–7.67 (d, 2H, *J* = 8.2 Hz), 7.60–7.58 (d, 2H, *J* = 8.2 Hz), 7.50–7.48 (d, 1H, *J* = 7.8 Hz), 6.83 (br s, 2H), 6.54 (s, 1H), 6.39–6.37 (d, 1H, *J* = 7.2 Hz), 5.27–5.26 (m, 2H), 1.82 (s, 6H), ^{13}C NMR (125 MHz, Acetone-d$_6$): 181.7, 170. 8, 162.3, 161.0, 158.2, 145.2, 143.6, 136.0, 134.8, 132.6, 132.30, 132.29, 131.03, 131.01, 130.8, 130.3, 129.32, 129.30, 129.2, 128.3, 126.3, 126.2, 115.6, 108.7, 106.5, 95.8, 50.6, 12.3, 10.7. HRMS m/z calculated for $C_{29}H_{22}F_6N_3O_2$, 558.1617; found [M+H]$^+$ 558.1601.

(*E*)-2-((2,5-Dimethyl-1-(2-(trifluoromethyl)phenyl)-1*H*-pyrrol-3-yl)methylene)-5-(pyridin-4-ylmethyl)-1*H*-pyrrolo[3,2-c]pyridine-3,4(2*H*,5*H*)-dione (64).

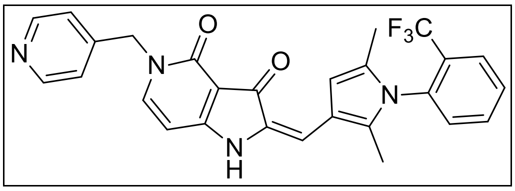

Yellow powder (0.020 g, 89%), mp 215–240 ºC. ^{1}H NMR (500 MHz, Acetone-d$_6$): δ 9.30 (s, 1H, -N*H*), 8.44–8.43 (d, 2H, *J* = 4.5, 1.5 Hz), 7.93–7.91 (d, 2H, *J* = 6.9 Hz), 7.93–7.90 (t, 1H, *J* = 8.0 Hz), 7.86–7.83 (t, 1H, *J* = 7.6 Hz), 7.77–7.75 (t, 1H, *J* = 7.7 Hz), 7.72–7.71 (d, 1H, *J* = 7.2 Hz), 7.42–7.42 (d, 1H, *J* = 7.85 Hz), 7.19–7.18 (d, 2H, *J* = 5.9 Hz), 6.62 (br s, 1H), 6.43 (s, 1H), 6.28–6.23 (d, 1H, *J* = 7.2 Hz), 4.11–4.07 (m, 1H), 1.82 (s, 3H), 1.74–1.68 (m, 3H). ^{13}C NMR (125 MHz, Acetone-d$_6$): 180.5, 179.3, 170.3, 161.0, 158.0, 150.8, 156.7, 145.3, 144.6, 135.0, 134.9, 134.7, 134.3, 131.7, 131.0, 131.3, 130.8, 130.3, 129.1, 115.6, 114.0, 106.9, 105.6, 103.6, 93.0, 50.2, 12.3, 10.7. HRMS m/z calculated for $C_{27}H_{22}F_3N_4O_2$, 491.1696; found $[M+H]^+$ 491.1686.

III. Physicochemical Evaluation

Calculated Parameters: Theoretical physicochemical values were calculated using the ACD/Labs Release 9.0 software (Advanced Chemistry Development, Toronto).

Solubility Estimation: Aqueous solubility was estimated by nephelometry. Compound in DMSO was spiked into either water, pH 6.5 phosphate buffer or 0.01N HCl (approx. pH 2.0) with the final DMSO concentration being 1%. Samples were then analyzed by Nephelometry to determine the solubility range as described previously.[1]

IV. Metabolism and Efficacy.

Experimental Methods

In Vitro Metabolic stability

Assays at Dundee

Test compound (0.5 μM) was incubated with female CD1 mouse liver microsomes (Xenotech LLC™; 0.5 mg/mL 50 mM potassium phosphate buffer, pH 7.4) and the reaction started with addition of excess NADPH (8 mg/mL 50 mM potassium phosphate buffer, pH 7.4). Immediately, at time zero, then at 3, 6, 9, 15 and 30 minutes an aliquot (50 μL) of the incubation mixture was removed and mixed with acetonitrile (100 μL) to stop the reaction. Internal standard was added to all samples, the samples centrifuged to sediment precipitated protein and the plates then sealed prior to UPLCMSMS analysis using a Quattro Premier XE (Waters corporation, USA).

XLfit (IDBS, UK) was used to calculate the exponential decay and consequently the rate constant (k) from the ratio of peak area of test compound to internal standard at each timepoint. These values were then used to calculate the in vitro intrinsic clearance value ($CL_{int,}$ μL/min/mg microsomal protein).

Verapamil (0.5 μM) was used as a positive control to confirm acceptable assay performance.

Assays at Monash

Metabolic stability was performed by incubating test compounds individually (1 μM) at 37 ºC with liver microsomes (BD Gentest, Discovery Labware Inc., Woburn, Massachusetts). The metabolic reaction was initiated by the addition of an NADPH-regenerating system and aliquots quenched at various time points by the addition of acetonitrile. Two additional samples incubated with NADPH and UDPGA (the co-factor for glucuronidation) were also included in the incubation for the qualitative assessment of the potential for glucuronide formation. The relative loss of parent compound and formation of metabolic products was monitored by LC/MS using a Waters/Micromass LCT mass spectrometer. The first order rate constant for substrate depletion was determined by fitting the data to an exponential decay function and these values were used to calculate the *in vitro* intrinsic clearance value ($CL_{int,}$ μL/min/mg microsomal protein).

V. Design and Results of the in vitro & in vivo Studies.

A. Drug inhibition of in vitro cultured *P. falciparum* parasite:[2] All in vitro assays were carried out twice independently. In vitro activity against the erythrocytic stages of *P. falciparum* was determined by using a ^{3}H-hypoxanthine incorporation assay,[5-6] using the chloroquine and pyrimethamine resistant K1 strain[7] and the standard drugs chloroquine (Sigma C6628) and artemisinin (Arteannuin, Qinghaosu; Sigma 36,159- 3). Compounds were dissolved in DMSO at 10 mg/mL and added to parasite cultures incubated in RPMI 1640 medium without hypoxanthine, supplemented with HEPES (5.94 g/L), $NaHCO_3$ (2.1 g/L), neomycin (100 U/mL), Albumax^{R} (5 g/L), and washed human red cells A+ at 2.5% haematocrit (0.3% parasitaemia). Serial doubling dilutions of each drug were prepared in 96-well microtiter plates and incubated in a humidified atmosphere at 37 °C; 4% CO_2, 3% O_2, 93% N_2. After 48 h, 50 µL of ^{3}H-hypoxanthine (= 0.5 µCi) was added to each well of the plate. The plates were incubated for an additional 24 h under the same conditions. The plates were then harvested with a Betaplate cell harvester (Wallac, Zurich, Switzerland), and the red blood cells were transferred onto a glass fiber filter and then washed with distilled water. The dried filters were inserted into a plastic foil with 10 mL of scintillation fluid and were counted in a Betaplate™ liquid scintillation counter (Wallac, Zurich, Switzerland); IC_{50} values were calculated from sigmoidal inhibition curves using Microsoft Excel.

B. In vitro cytotoxicity assay:[2] Assays were performed in 96-well microtiter plates, each well containing 100 μl of RPMI 1640 medium supplemented with 1% L-glutamine (200 mM) and 10% fetal bovine serum, and 4 x 10^{4} L-6 cells (a primary cell line derived from rat skeletal myoblasts). Serial drug dilutions of seven 3-fold dilution steps covering a range from 90 to 0.123 µg/mL were prepared. After 72 h of incubation the plates were inspected under an inverted microscope to assure growth of the controls and sterile conditions. 10 μL of Alamar Blue was then added to each well and the plates incubated for another 2 h. Then the plates were read with a Spectramax Gemini XS microplate fluorometer (Molecular Devices Cooperation, Sunnyvale, CA, USA) using an excitation wave length of 536 nm and an emission wave length of 588 nm. Data were analysed using the microplate reader software Softmax Pro (Molecular Devices Cooperation, Sunnyvale, CA, USA).

C. In vivo antimalarial efficacy studies (4 days treatment).[2]

In vivo antimalarial activity was assessed basically as previously described. Groups of three female NMRI mice (20–22 g) intravenously infected with 2 x 10^7 parasitized erythrocytes on day 0 with GFP-transfected *P. berghei* strain ANKA. .Unless otherwise indicated compounds were formulated in 100% DMSO, diluted 10-fold in distilled water and administered intraperitoneally or orally in a volume of 10 mL kg^{-1} on four consecutive days (4, 24, 48 and 72 h post infection). Parasitemia was determined on day 4 post infection (24 h after last treatment) by FACS analysis. Activity was calculated as the difference between the mean per cent parasitaemia for the control (n=5 mice) and treated groups expressed as a per cent relative to the control group. The survival time in days was also recorded up to 30 days after infection. A compound was considered curative if the animal survived to day 30 after infection with no detectable parasites. All protocols and procedures used in the current study were reviewed and approved by the local veterinary authorities of the Canton Basel-Stadt.

VI. Additional References.

1. Veber, D. F.; Johnson, S. R.; Cheng, H. Y.; Smith, B. R.; Ward, K. W.; Kopple, K. D. Molecular properties that influence the oral bioavailability of drug candidates. *J. Med. Chem.* **2002,** 45, 2615-2623.
2. Murugesan, D.; Mital, A.; Kaiser, M.; Shackleford, D. M.; Morizzi, J.; Katneni, K.; Campbell, M.; Hudson, A.; Charman, S. A.; Yeates, C.; Gilbert, I. H. Discovery and Structure-Activity Relationships of Pyrrolone Antimalarials. *J. Med. Chem.* **2013**, *56,* 2975-2990.
